# Decoding the prognostic significance of integrator complex subunit 9 (INTS9) in glioma: links to TP53 mutations, E2F signaling, and inflammatory microenvironments

**DOI:** 10.1186/s12935-023-03006-5

**Published:** 2023-08-03

**Authors:** Yu-Chieh Lin, Pei-Chi Chang, Dueng-Yuan Hueng, Shih-Ming Huang, Yao-Feng Li

**Affiliations:** 1 Department of Pathology and Laboratory Medicine, Taoyuan Armed Forces General Hospital, Taoyuan, 325 Taiwan, Republic of China; 2 Graduate Institute of Medical Sciences, National Defense Medical Center, Taipe, 114 Taiwan, Republic of China; 3 Graduate Institute of Life Sciences, National Defense Medical Center, Taipe, 114 Taiwan, Republic of China; 4 Department of Neurologic Surgery, Tri-Service General Hospital, National Defense Medical Center, Taipe, 114 Taiwan, Republic of China; 5 Department of Biochemistry, National Defense Medical Center, Taipe, 114 Taiwan, Republic of China; 6 Department of Pathology, Tri-Service General Hospital, National Defense Medical Center, Taipe, 114 Taiwan, Republic of China

**Keywords:** INTS9, Glioblastoma, Glioma, TCGA, CGGA, GLASS, GSEA, TMB, MSI, CIBERSORT, Single-cell sequencing, TP53

## Abstract

**Introduction:**

Gliomas, a type of brain neoplasm, are prevalent and often fatal. Molecular diagnostics have improved understanding, but treatment options are limited. This study investigates the role of INTS9 in processing small nuclear RNA (snRNA), which is crucial to generating mature messenger RNA (mRNA). We aim to employ advanced bioinformatics analyses with large-scale databases and conduct functional experiments to elucidate its potential role in glioma therapeutics.

**Materials and methods:**

We collected genomic, proteomic, and Whole-Exon-Sequencing data from The Cancer Genome Atlas (TCGA) and Chinese Glioma Genome Atlas (CGGA) for bioinformatic analyses. Then, we validated INTS9 protein expression through immunohistochemistry and assessed its correlation with P53 and KI67 protein expression. Gene Set Enrichment Analysis (GSEA) was performed to identify altered signaling pathways, and functional experiments were conducted on three cell lines treated with siINTS9. Then, we also investigate the impacts of tumor heterogeneity on INTS9 expression by integrating single-cell sequencing, 12-cell state prediction, and CIBERSORT analyses. Finally, we also observed longitudinal changes in INTS9 using the Glioma Longitudinal Analysis (GLASS) dataset.

**Results:**

Our findings showed increased INTS9 levels in tumor tissue compared to non-neoplastic components, correlating with high tumor grading and proliferation index. TP53 mutation was the most notable factor associated with upregulated INTS9, along with other potential contributors, such as combined chromosome 7 gain/10 loss, TERT promoter mutation, and increased Tumor Mutational Burden (TMB). In GSEA analyses, we also linked INTS9 with enhanced cell proliferation and inflammation signaling. Downregulating INTS9 impacted cellular proliferation and cell cycle regulation during the function validation. In the context of the 12 cell states, INTS9 correlated with tumor-stem and tumor-proliferative-stem cells. CIBERSORT analyses revealed increased INTS9 associated with increased macrophage M0 and M2 but depletion of monocytes. Longitudinally, we also noticed that the INTS9 expression declined during recurrence in IDH wildtype.

**Conclusion:**

This study assessed the role of INTS9 protein in glioma development and its potential as a therapeutic target. Results indicated elevated INTS9 levels were linked to increased proliferation capacity, higher tumor grading, and poorer prognosis, potentially resulting from TP53 mutations. This research highlights the potential of INTS9 as a promising target for glioma treatment.

**Supplementary Information:**

The online version contains supplementary material available at 10.1186/s12935-023-03006-5.

## Introduction

Brain neoplasms constitute a prevalent category of cancer, leading to significant morbidity and mortality on a global scale. According to the United States Central Brain Tumor Registry (CBTRUS), histological analysis reveals that diffuse glioma constitutes 68.5% of primary cerebral malignancies, with glioblastoma being the most lethal subtype [1]. Following the revisions made in the 4th World Health Organization (WHO) Classification in 2016 [2] and a series of subsequent cIMPACT-NOW updates [3–10], molecular characteristics have assumed a pivotal role in the predictive determination and therapeutic approach. Key molecular markers encompass IDH1/2 [11, 12], 1p19q [8], homozygous deletions of CDKN2A/B [5], EGFR amplification [9], TERT promoter mutations [9], chromosome 7/10 alterations [9], and MGMT promoter methylation status [13]. The new glioma classification system, published in 2021 [14], integrated these suggestions. Besides gene status, the significance of tumor methylation patterns as crucial diagnostic tools has been acknowledged [15]. Even with advancements in molecular diagnostics, the available treatment choices for brain tumors remain restricted. Currently, the standard of care involves gross surgical resection, followed by chemotherapy and radiotherapy, which can extend survival to approximately 16 months [16–18]. Temozolomide (TMZ) is the conventional first-line chemotherapeutic agent; however, gliomas often develop resistance to TMZ, resulting in a dismal prognosis [19]. In these decades, substantial efforts have been directed toward identifying novel therapeutic targets for treating brain tumors. While numerous pertinent clinical trials have been conducted [20], the results have not met expectations. Consequently, identifying novel therapeutic targets is crucial for expanding chemotherapeutic options [21].

INTS9, also known as Integrator Complex Subunit 9, is part of the core cleavage module consisting of INTS4/INTS9/INTS11 involved in processing small nuclear RNA (snRNA) molecules [22, 23]. It is a subunit of the Integrator complex, which comprises 15 subunits (including INTS6L), and plays a crucial role in the 3’ end processing of snRNAs [24, 25]. By participating in the biogenesis of snRNAs, INTS9 contributes to the proper functioning of the spliceosome, a cellular machinery responsible for the accurate splicing of precursor messenger RNA (pre-mRNA) molecules [25]. This process is essential for generating mature messenger RNA (mRNA), which is subsequently translated into functional proteins [25]. In this investigation, our objective was to delineate the role of INTS9 in glioma. To achieve this, we employed an array of advanced bioinformatics analyses utilizing large-scale databases and conducted functional experiments using small interfering RNA targeting the INTS9 gene (siINTS9) to elucidate its involvement in glioma.

## Materials and methods

### Data collection for bioinformatics analyses

The analyzed data set was divided into five parts. First, the primary INTS9 bioinformatics study employed the Cancer Genome Atlas (TCGA) transcriptome (https://portal.gdc.cancer.gov/), which included transcriptomes (mRNA), methylation data (illumina human methylation 450 K), and clinical variables like gender, age, histology, tumor grade, overall survival, and vital status. We recruited 690 cases (Supplementary 1 A), but these followed an old classification system [26], so we reclassified cases into three subgroups following the 2021 WHO classification [14], including IDH wildtype, IDH mutant astrocytoma, and oligodendroglioma. We also gathered 892 cases with Whole-Exome Sequencing (WES) to compute tumor mutation burden (TMB) for further study (Supplementary 2). Glioma ATAC sequencing data (https://gdc.cancer.gov/about-data/publications /ATACseq-AWG, retrieved Sep. 2022) was used to understand the association between INTS9 and chromatin accessibility. There were 13 glioma cases with ATAC sequencing data available for further analysis (Supplementary 1B). To correlate the INTS9 with the chromosomal aberrations, we collected information on EGFR amplification, chromosome 7 gain/10 loss, TERT promoter mutation, and CDKN2A/2B deletion from cBioPortal (https://www.cbioportal.org/, retrieved Sep. 2022). Second, we used the proteomics database from TCGA PDC (https://proteomic.datacommons.cancer.gov/pdc/, retrieved Sep. 2022) to confirm the transcriptome expression. The dataset contained 110 proteomic cases, including 100 glioblastomas and 10 normal controls (Supplementary 1 C), with clinical information such as patient age, gender, diagnosis, and grade. Third, for the tumor heterogeneity and immune cell analyses, we utilized TCGA, the Chinese Glioma Genome Atlas (CGGA, http://www.cgga.org.cn/, retrieved Sep. 2022), and Glioma Longitudinal AnalySiS (GLASS) data (https://www.synapse.org/#!Synapse: syn17038081/wiki/585,622, retrieved Sep. 2022). Here, we collected 1012 patient records from CGGA (Supplementary 1D) and 423 samples from GLASS (Supplementary 1E). In the fourth part, we used the GLASS dataset, which contained 176 cases with paired samples (130 IDH wildtype astrocytoma, 28 cases of IDH mutant astrocytoma, and 10 cases of oligodendroglioma) for longitudinal analysis. Finally, we utilized two single-cell sequencing datasets from the Gene-Expression Omnibus (GEO) databases: one for IDH wildtype (GSE131928, https://www.ncbi.nlm.nih.gov/geo/query/acc.cgi?acc=GSE131928, retrieved Sep. 2022) and another for IDH mutant (GSE89567, from https://www.ncbi.nlm.nih.gov/geo/query/acc.cgi?acc=GSE89567, retrieved Sep. 2022).

### Transcriptome, proteomics, and single-cell sequencing data processing

We conducted bioinformatics analyses using R (R version 4.1.0, www.r-project.org) and relevant R packages. First, we converted the downloaded gene expression data unit, RPKM (Reads Per Kilobase per Million), to TPM (Transcripts Per Million). We then employed the ggpubr and limma packages to analyze the mRNA regression, and differentially expressed genes, using an adjusted P-value < 0.05 as the threshold. Next, we carried out Kaplan-Meier survival and COX-survival analyses to assess the effect of INTS9 on prognosis. To examine the impact of genomic stability, we downloaded whole-exon sequencing data to calculate TMB using the “maftools” package. We retrieved Microsatellite Instability (MSI) data from the landmark study by Sameek Roychowdhury’s team [27]. For single-cell sequencing, We analyzed the GSE131928 and GSE89567 datasets on the BBrowser platform [28].

### Gene Set Enrichment Analysis (GSEA)

The Gene Set Enrichment Analysis (GSEA, https://www.gseamsigdb.org/gsea/index.jsp, accessed Sep. 2022) [29, 30] is an effective computational method for analyzing gene expression data to identify altered signaling pathways by comparing two different groups. First, we used GSEA to examine the differences in signaling between high and low-INTS9 expression groups (using the median as the cut-off value) in TCGA glioma datasets. We employed the “hallmark gene-sets v7.5” (accessed Sep. 2022) as a reference and kept other parameters at their default values. In the functional validation of GSEA analysis, we compared the mRNA sequencing between GBM8401 cells with scrambled small interfering RNA (siRNA) and GBM8401 cells treated with small interfering RNA targeting the INTS9 gene (siINTS9).

### Twelve-cell state and CIBERSORT analyses

Twelve-cell state prediction and CIBERSORT (https://cibersort.stanford.edu/) analyses are developed by Roel G W Verhaak [32] and Newman et al. at the Alizadeh Lab [31]. The Twelve-cell state deconvolution enables us to identify the critical 12-cell components in each glioma sample [32]. CIBERSORT calculations allow us to further evaluate the abundance of various immune cell types within a mixed population using gene expression data. We used bulk transcriptome data from the TCGA, CGGA, and GLASS databases as input to calculate the percentages of 12 cellular components and 22 different immune cells for each case, using a significance threshold of p < 0.05. Next, investigated the association between these proportions with INTS9 expression.

### Tissue microarray and immunohistochemistry

First, we acquired glioma tissue microarrays (GL1001a) from Biomax, Inc. (https://www.biomax.us/), which included tissue sections and clinical information. All human tissue specimens were collected with informed consent from donors through US Biomax, Inc. The tissue array contained 68 adult glioma cases (7 for Grade 1, 43 for Grade 2, 10 for Grade 3, and 8 for Grade 4) and 10 normal brain tissues. The core size was 1.5 mm in diameter with a tissue thickness of 5 μm. We used the Ventana Benchmark®XT immunostainer for immunohistochemical staining to ensure consistent staining. Before staining, the formalin fixed paraffin embedded tissue sections were heated in the pressure cooker at 125 °C for 30 min for antigen retrieval (0.01 M sodium citrate, pH 6.2) and washed in phosphate-buffered saline (PBS) three times and five minutes each. Following this, we loaded the slides onto the autostainer according to the manufacturer’s instructions. Primary antibodies used included INTS9 (Atlas antibodies #HPA051615), KI67 (Abcam #ab15580), and P53 (cell signaling # 2524). We used Roche Diagnostics OptiView DAB IHC Detection Kit for the secondary antibodies. Positive and negative controls were utilized to assess the antibodies’ binding capabilities. We observed strong staining in the positive control but not in the negative control.

### Scoring of the immunohistochemistry

Following previously published methods [32, 33], we examined and scored stained slides using an automated semi-quantitative system. First, stained slides were digitally scanned, and the TIFF files were exported as 10x images. Next, we utilized ImageJ Fiji with macro coding to automatedly quantify the entire area, following the protocol described in earlier work [33]. Staining areas were assigned scores from 0 to 3, representing negative to strongly positive staining. We estimated tumor-stained area fractions (0-100%) and an Immunoscore (ranging from 0 to 300) based on the sum of each intensity multiply its corresponding staining percentage. Finally, we employed an ANOVA to assess the significance of immunostaining scores to clinical parameters.

### Human glioma cell lines and Lysate Preparation

We used the following glioma cell lines for functional validation: U87MG, U118MG, LN229, LNZ308, GBM8401, SVGp12, and NHA. These were maintained in DMEM supplemented with 100 units/mL of penicillin, 10% fetal bovine serum (FBS), and 100 µg/mL of streptomycin. Cultures were then kept at 37 °C in an incubator with 5% CO_2_. We assessed INTS9 protein expression via Western blotting. Actin, GAPDH, and β-actin served as internal controls. Additionally, we used normal brain lysate purchased from Biocompare (MBS537208, San Francisco, CA, USA) as a control.

### Western blot analysis

We washed the cell lines with PBS and lysed them in RIPA buffer (100 mM Tris-HCl in pH 8.0, 150 mM NaCl, 0.1% SDS, and 1% Triton 100). We separated the protein lysates (20–40 µg, based on the concentrations) using 10% SDS-PAGE and analyzed them through immunoblotting with antibodies against polyclonal rabbit anti-GAPDH (sc-47,724, Santa Cruz), anti-INTS9 (Atlas antibodies #HPA007674), and the monoclonal mouse anti-ACTN (sc-17,829, Santa Cruz). We repeated the experiment and quantified the images using the ImageJ software (National Institutes of Health, USA).

### RNA extraction and real-time PCR

We prepared a total RNA extract using TRIzol™ Reagent (Thermo Fisher Scientific, USA), followed by reverse transcription with Tetro™ Reverse Transcriptase (Bioline, Taunton, MA, USA). We conducted qRT-PCR with Fast Plus EvaGreen qPCR Master Mix (Biotium, Fremont, CA, USA) and the StepOne™ Real-Time PCR (Thermo Fisher Scientific, USA). Then, performed thermocycling using 0.25 µM of each primer (PrimerBank) and 2.5 µL of cDNA diluent. The PCR process incorporated a 95°C denaturation for two minutes, succeeded by 40 cycles of touch-down PCR, consisting of a 5-second duration at 95°C and a 30-second annealing phase at 60°C. The primers used included: for INTS9, 5’-CCCACTGCAAACCCAGATGGAA-3’ (forward) and 5’-TAGGC ACTCCAGGAGG TCATAG-3’ (reverse); for GAPDH, 5’-GCACCGTCAAGGCTGAGAAC-3’ (forward) and 5’-ATGGTGGTGAAGACGCCAGT-3’ (reverse).

### Transfection of scrambled siRNA and siINTS9 into Glioma Cell Lines

In this study, we purchased scrambled siRNA and siINTS9 from Dharmacon (Lafayette, product ID, M-020275-00-0005). In 6-well plates, cells were transfected with 25 nM scrambled siRNA and siINTS9 (Dharmacon, USA) using DharmaFECT 1 Transfection Reagent. After placing cells into the 6-well plates, cells were incubated with 5 × 10^4^ cells per well overnight at 37 °C. Then, cells were harvested by adding 200 µL of 10% FBS DMEM and 100 µL of 0.05% trypsin. Ultimately, a Western blot analysis was conducted to assess EMT phenotype (N-cadherin), cell cycle (cyclin D1), autophagy (LC3I, LC3II, P62), mitochondrial biogenesis (mtTFA), and the expression of reactive oxygen species-associated marker (Nrf2) in cells after scrambled siRNA and siINTS9 transfection.

### Cell-cycle, apoptosis analysis, and fluorescence-activated cell sorting (FACS)

For the cell cycle analysis, the LNZ308, U87MG, and GBM8401 cells were transfected with 25 nM siINTS9 for 72 h, after which they were collected, fixed using 70% ethanol, washed with PBS/1% FBS, and treated with 10 µg/mL RNAse A and 50 µg/mL propidium iodide in PBS, along with 1% Tween 20 for 30 min at 37 °C in the dark. To assess the occurrence of apoptosis utilizing the PE-annexin V Apoptosis Detection Kit, following the guidelines provided by BD Biosciences. Flow cytometry analysis was carried out using a FACS Calibur flow cytometer (BD Biosciences, USA). The Cell Quest Pro software program (BD Biosciences, USA) was employed to determine the proportions in the cell cycle phases.

### Measure the level of reactive oxygen species (ROS) in the cell cytosol

To detect the production of ROS in the cytoplasm, we plated cells in six-well plates and treated them with scrambled siRNA or siINTS9 to assess ROS production by the cells. After 3 h of treatment with the substance, living cells were stained with 10 µM DCFH-DA (Sigma Aldrich) for 10 min at 37 °C and then incubated for 3 h. Flow cytometry was then used to determine the percentage of stained cells.

### Analysis of RNA sequences from functional validation

Transcriptomes of GBM8401 cells were obtained from six separate experiments, comprising three control samples with scrambled siRNA and another three samples with siINTS9. Total RNA extraction was carried out utilizing TRIzol (Invitrogen) and analyzed at 260 nm, 280 nm, and 230 nm, with RNA Integrity Number (RIN) values for quality control. The RIN values for the six samples ranged from 9.7 to 10. We then converted mRNA into 300–500 bp cDNA fragments through reverse transcription and attached adapters to both ends using the mRNA Library Kit. RNA sequencing was performed on the NextSeq 550 System with the adapter fragment connected to a flow cell and amplified via Bridge PCR. A bioinformatics pipeline from the Instrument Center of the National Defense Medical Center supplied the expression data in counts, RPKM, and TPM formats for further GSEA analysis.

## Results

### Result 1: INTS9 increased in the tumor component and played a prognostic role in glioma

**Fig. 1:**
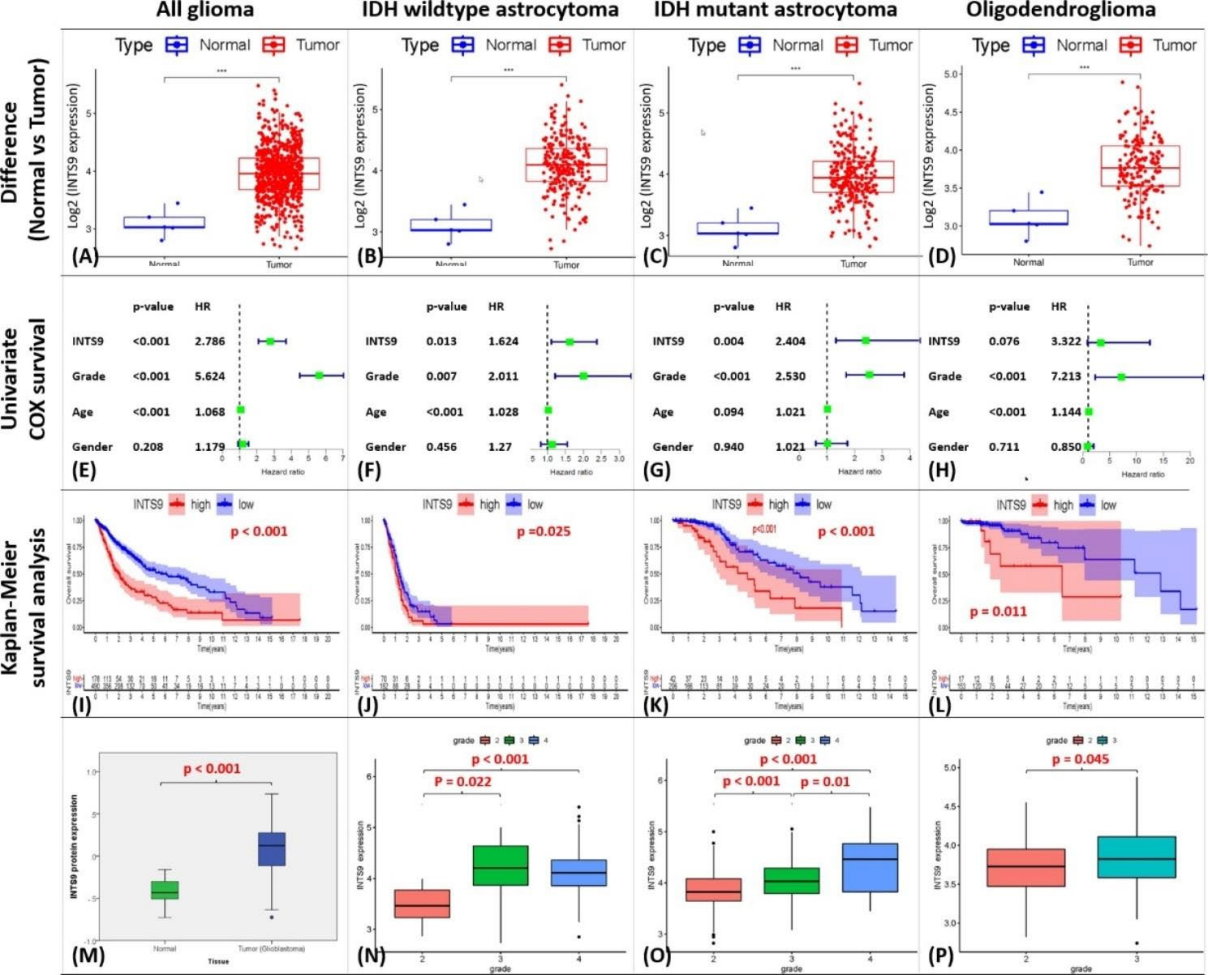
Investigation of INTS9 mRNA and proteomics expression from TCGA dataset using bioinformatics analyses. (**A-D**) Increased levels of INTS9 can be observed within tumors compared to normal components across a wide range of gliomas and all related subgroups. (**E-H**) COX survival analysis results and (**I-L**) Kaplan-Meier survival analysis findings collectively suggest that INTS9 has an unfavorable prognostic impact in astrocytoma groups. (**M**) Proteomics examination further revealed a markedly elevated expression of INTS9 in tumor components. (**N-P**) Moreover, all investigated subgroups identified a strong association between INTS9 and higher tumor grades

The examination of bioinformatics data from the TCGA dataset demonstrated a significant elevation in INTS9 levels within the tumor compared to normal components across pan-glioma and all subgroups (Fig. 1A-D). A COX-survival analysis found that elevated INTS9 levels were significantly associated with a higher hazard ratio in IDH-wildtype and IDH-mutant astrocytoma (Fig. 1E-G), while only exhibiting a borderline association in oligodendroglioma (Fig. 1H). The Kaplan-Meier survival analysis indicated a worse prognosis for individuals with high INTS9 expression in all groupings (Fig. 1I-L). In order to corroborate whether the protein expression exhibited a similar pattern, TCGA proteomic data for INTS9 was also examined. Consistent with the transcriptome findings, INTS9 protein expression was significantly elevated in tumor components compared to normal (Fig. 1M). Furthermore, analyses of clinical factors revealed that increased INTS9 expression was significantly correlated with higher grading across all groups (Fig. 1N-P).

### Result 2: INTS9 correlated with tumor grading, P53 status, and proliferation index

**Fig. 2:**
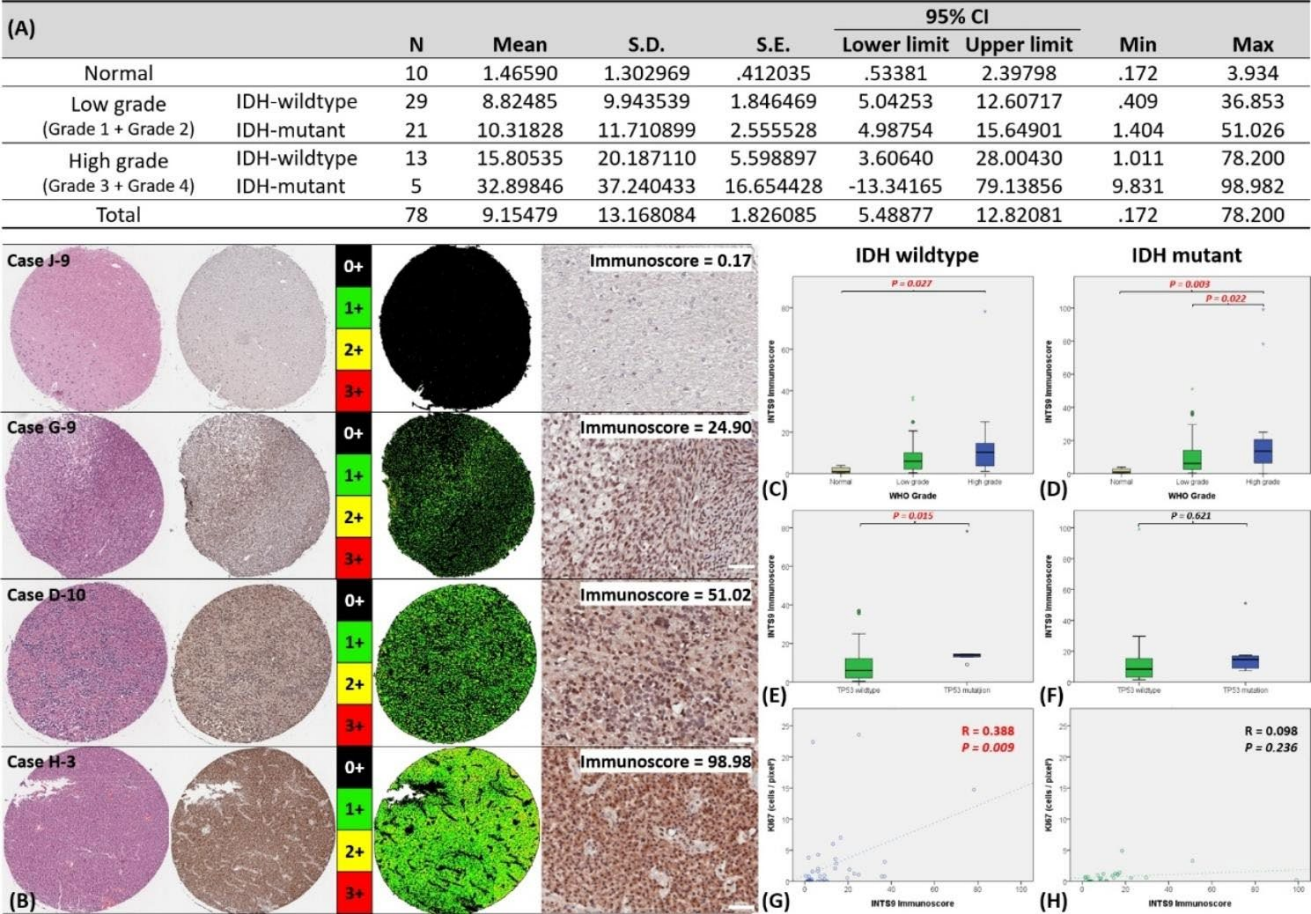
Quantify the INTS9 immunohistochemistry and the correlation with the prognostic factors. (**A**) Immunohistochemistry was used to analyze INTS9 protein expression in 78 clinical cases. (**B**) Display of immunohistochemistry and corresponding Immunoscore quantification for four selected cases. (**C-D**) The higher INTS9 Immunoscore was associated with increased tumor grades in both astrocytoma groups. (**E**) Elevated INTS9 was relevant to mutant P53 status and (**G**) KI67 labeling index in IDH wildtype astrocytoma (**F** and **H**) but not significant in IDH mutant group

Utilizing immunohistochemistry, we examined INTS9 protein expression in tissues from 78 clinical cases, as detailed in (Fig. 2A). ImageJ software evaluated all images, classifying staining intensities on a scale from 0 (negative) to 3+ (strongly positive). Subsequently, we determined the percentage of the stained area (0-100%) for each intensity and calculated an Immunoscore (0-300) by multiplying the intensity with the corresponding fraction and summing the results, as illustrated in (Fig. 2B) and (Supplementary 3). The Immunoscore was correlated with clinical prognostic markers, such as age, gender, tumor grade, P53 status, and the KI67 proliferation index. In both groups, our findings revealed a connection between higher INTS9 Immunoscore and increased tumor grades (Fig. 2C-D). Furthermore, we observed a significant relationship between elevated INTS9 expression and mutant P53 status (Fig. 2E) and the KI67 labeling index (Fig. 2G) in IDH wildtype astrocytoma. However, this was insignificant in the IDH mutant group (Fig. 2F and H).

### Result 3: The potential upstream regulators of INTS9 expression encompass driven mutation, chromosomal structure abnormalities, genomic stability, and epigenetics

**Fig. 3:**
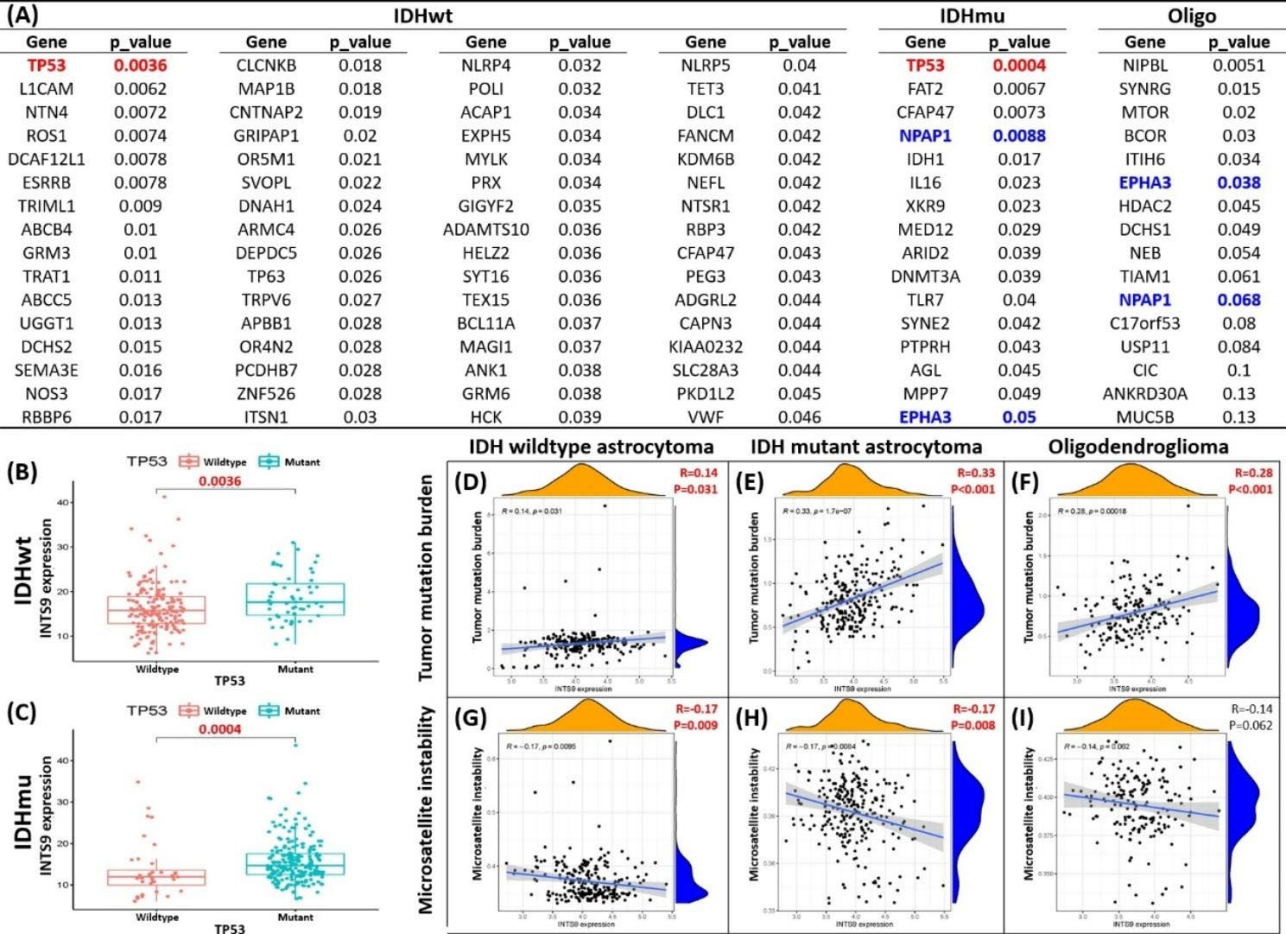
Utilizing the TCGA dataset and WES to identify the upstream factors that impact INTS9 elevation. (**A**) TP53 was significantly related to INTS9 in both IDH wildtype and mutant astrocytoma. (**B-C**) The box plots visualized the association between INTS9 and TP53 status. (**D-F**) Additionally, INTS9 positively correlated with TMB, but (**G-I**) negatively correlated with MSI.

**Fig. 4:**
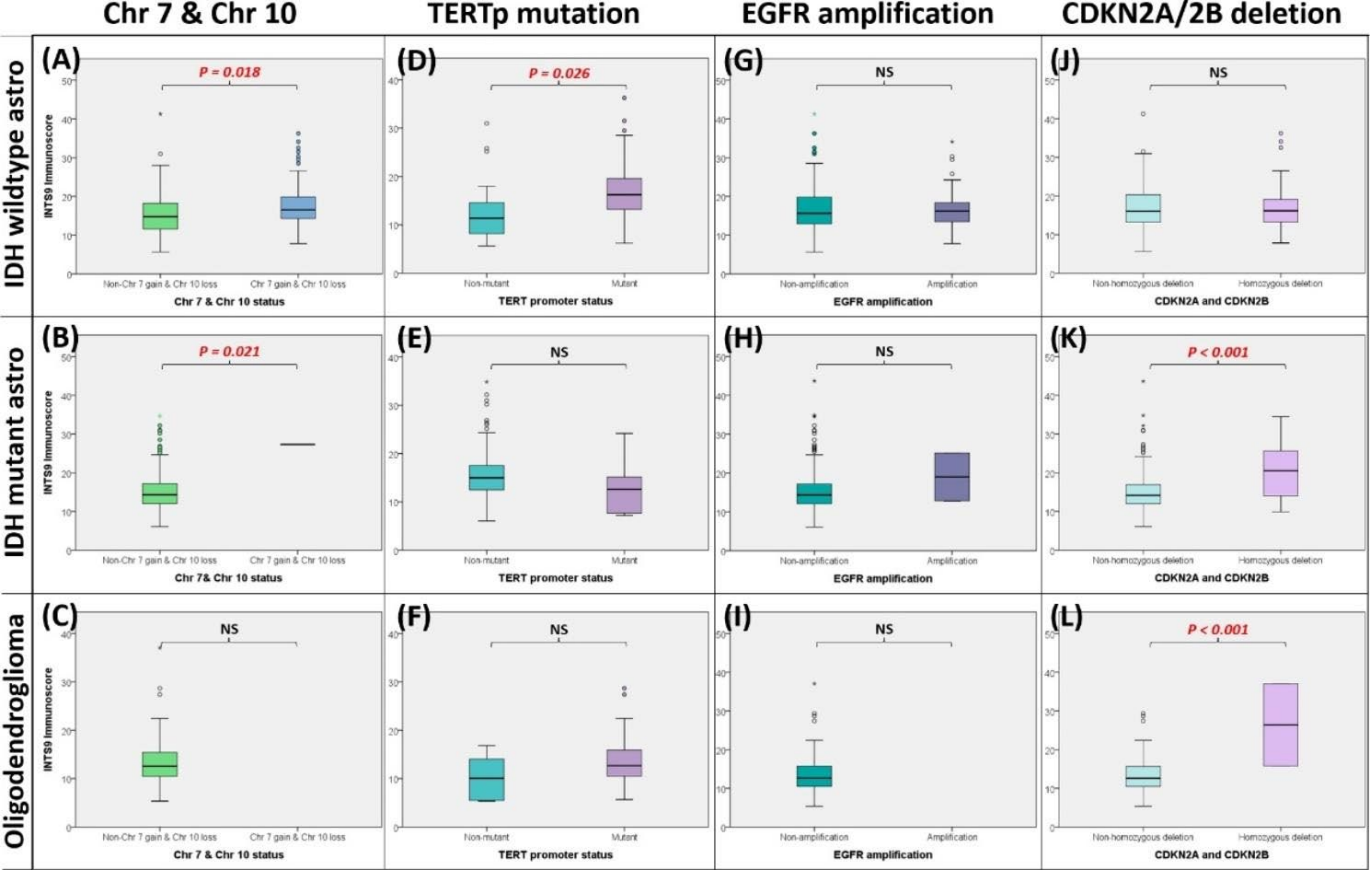
(**A-C**) Elevated INTS9 levels were linked to gains in chromosome 7 and losses in chromosome 10 in both IDH wildtype and mutant astrocytoma. (**D-F**) TERT promoter related only to IDH wildtype astrocytoma, (**G-I**) while EGFR amplification had no connection to INTS9 expression. (**J-L**) INTS9 expression associated with CDKN2A/2B deletion in IDH mutant astrocytoma and oligodendroglioma. NS: non-significant

**Fig. 5:**
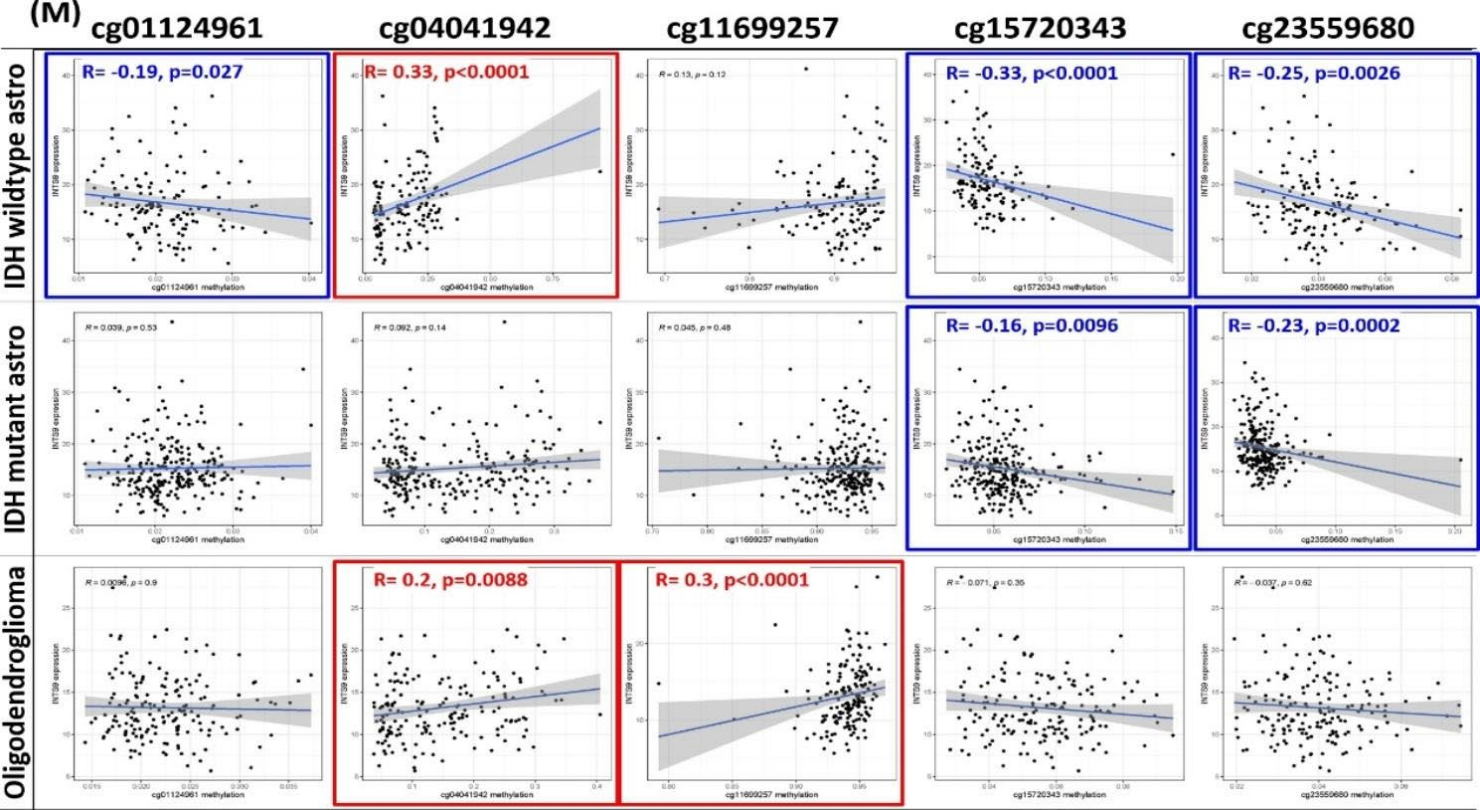
**Potential epigenetic regulators show slight differences across subtypes**. For IDH-mutant astrocytoma, cg15720343 and cg23559680 inversely regulate, while cg04041942 and cg11699257 have a positive relationship in oligodendroglioma. In IDH wildtype astrocytoma, methylation of specific sites suppresses or correlates positively with INTS9 expression. *Red signifies positive correlation, blue signifies negative

To verify the association between INTS9 and TP53, we scrutinized the transcriptome and WES utilizing the TCGA dataset. By analyzing 14,733 genes and their mutation status [33], we pinpointed genes correlated with INTS9 expression. Notably, TP53 emerged as the most significantly related gene in both IDH wildtype and mutant astrocytoma cohorts (Fig. 3A-C), in alignment with prior immunohistochemistry findings (Fig. 2E). Within the IDH mutant astrocytoma and oligodendroglioma groups, EPHA3 and NPAP1 surfaced as recurring genes potentially connected to the upregulation of INTS9. In terms of genomic stability, INTS9 displayed a positive correlation with TMB across all subgroups (Fig. 3D-F) and a negative correlation with MSI in both astrocytoma groups (Fig. 3G-H), exhibiting a marginal association in oligodendroglioma (Fig. 3I). Subsequently, DNA structure abnormality may also be involved beyond gene mutations and genomic stability. Our observations revealed that elevated INTS9 levels corresponded to the combined chromosome 7 gain/chromosome 10 loss status in IDH wildtype and mutant astrocytoma (Fig. 4A-C). In contrast, the TERT promoter was pertinent exclusively to IDH wildtype astrocytoma (Fig. 4D-F). However, extrachromosomal DNA (**EGFR amplification**) showed no connection to INTS9 expression within subgroups (Fig. 4G-I). The status of CDKN2A/CDKN2B homozygous deletion linked to the INTS9 expression was found only in the IDH mutant glioma, including IDH mutant astrocytoma and oligodendroglia (Fig. 4J-L). Lastly, the INTS9 expression might also be influenced by epigenetic regulation. Our investigations have revealed there were 13 CpG islands found located in the gene INTS9, and these methylation statuses potentially influence its expression. Correlational analyses revealed that 8 out of 13 were not associated with INTS9 expression, specifically cg00459975, cg01188976, cg03363242, cg05314271, cg07267296, cg10947480, cg14143435, and cg14442394. Conversely, the methylation status of the other 5 CpG islands (cg01124961, cg04041942, cg11699257, cg15720343, and cg23559680) seemed to affect INTS9 expression, showed as (Fig. 5). Briefly, the influence of potential epigenetic regulators varied slightly among different subtypes. In IDH-mutant astrocytoma, cg15720343 and cg23559680 functioned as primary inverse regulators, whereas cg04041942 and cg11699257 acted positively associated with oligodendroglioma. In contrast, in IDH wildtype astrocytoma, methylation of cg01124961, cg15720343, and cg23559680 led to the suppression of INTS9 expression, whereas methylation of cg04041942 positively correlated INTS9 expression.

### Result 4: Elevated INTS9 expression linked to increased cell proliferation and inflamed microenvironment

**Fig. 6:**
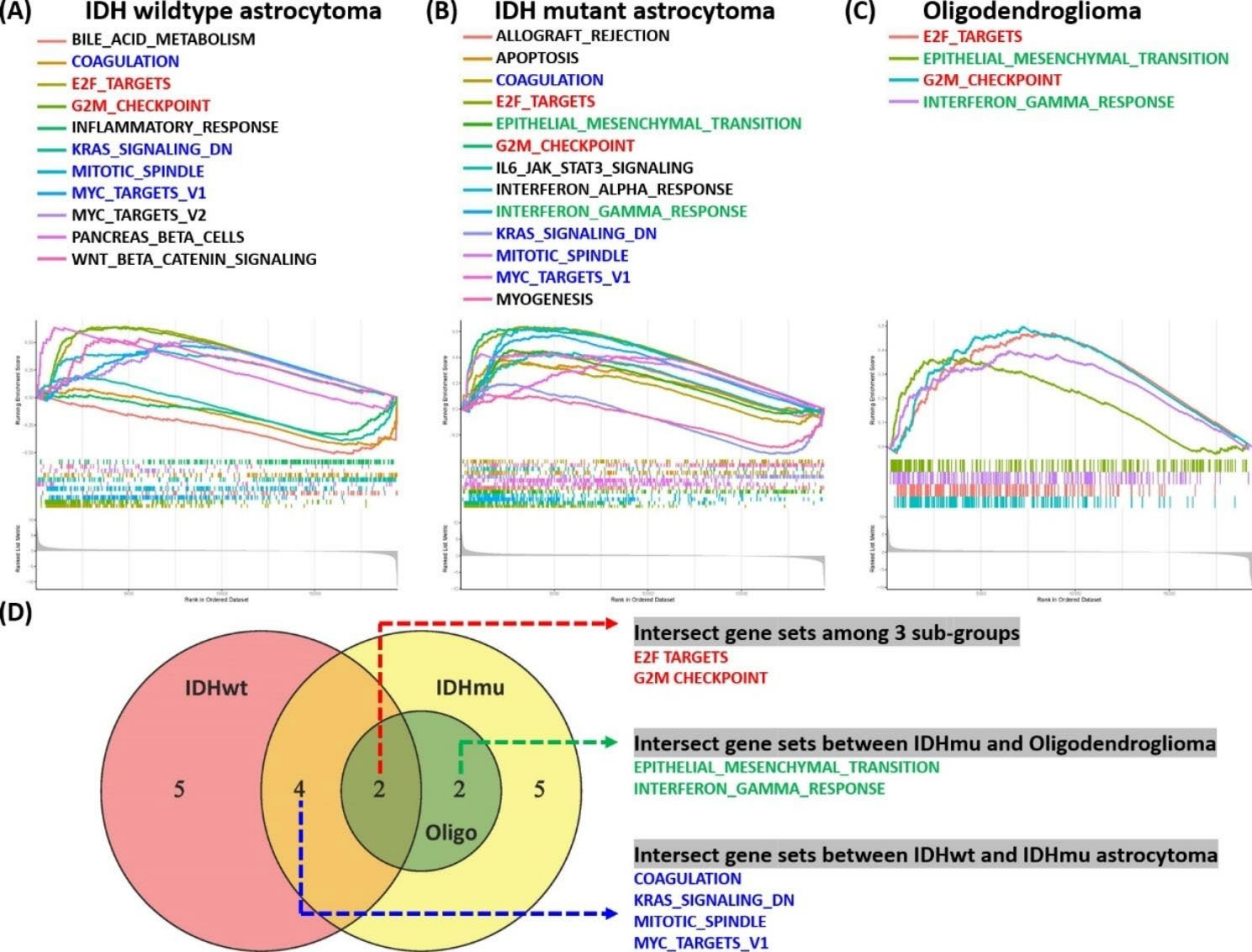
Utilizing GSEA to investigate TCGA datasets. (**A-C**) Two common gene sets, E2F_TARGETS and G2M_CHECKPOINT, were linked to cell proliferation and supported the INTS9-KI67 connection. (**D**) Additional overlapping gene sets in astrocytoma and IDH mutant subgroups were found, some related to cell growth and others to inflammatory cell migration

To comprehend the changes in signaling upon the elevation of INTS9 expression, we employed GSEA to examine the TCGA datasets further. The findings disclosed the modified signaling pertinent to increased INTS9 expression in each subgroup (Fig. 6A-C). Within the gene set lists, we pinpointed two common gene sets across these subgroups, namely E2F_TARGETS and G2M_CHECKPOINT, which are linked to cell proliferation and corroborate the previous connection between INTS9 and KI67 expression (Fig. 2G). In the case of astrocytoma groups, additional overlapping gene sets were found, including KRAS_SIGNALING_DN, MITOTIC_SPINDLE, MYC_TARGETS_V1, and COAGULATION (Fig. 6D), with the initial three sets being related to cell growth as well. Regarding IDH mutant astrocytoma and oligodendroglioma, two other shared gene sets were identified, EPITHELIAL_MESENCHYMAL_TRANSITION and INTERFERON_GAMMA_RESPONSE, which are linked to cellular migration and inflammation (Fig. 6D).

### Result 5: In the functional validation, the downregulation of INTS9 impacted the proliferation and cell cycles of GBM8401 but not the other cell lines

**Fig. 7:**
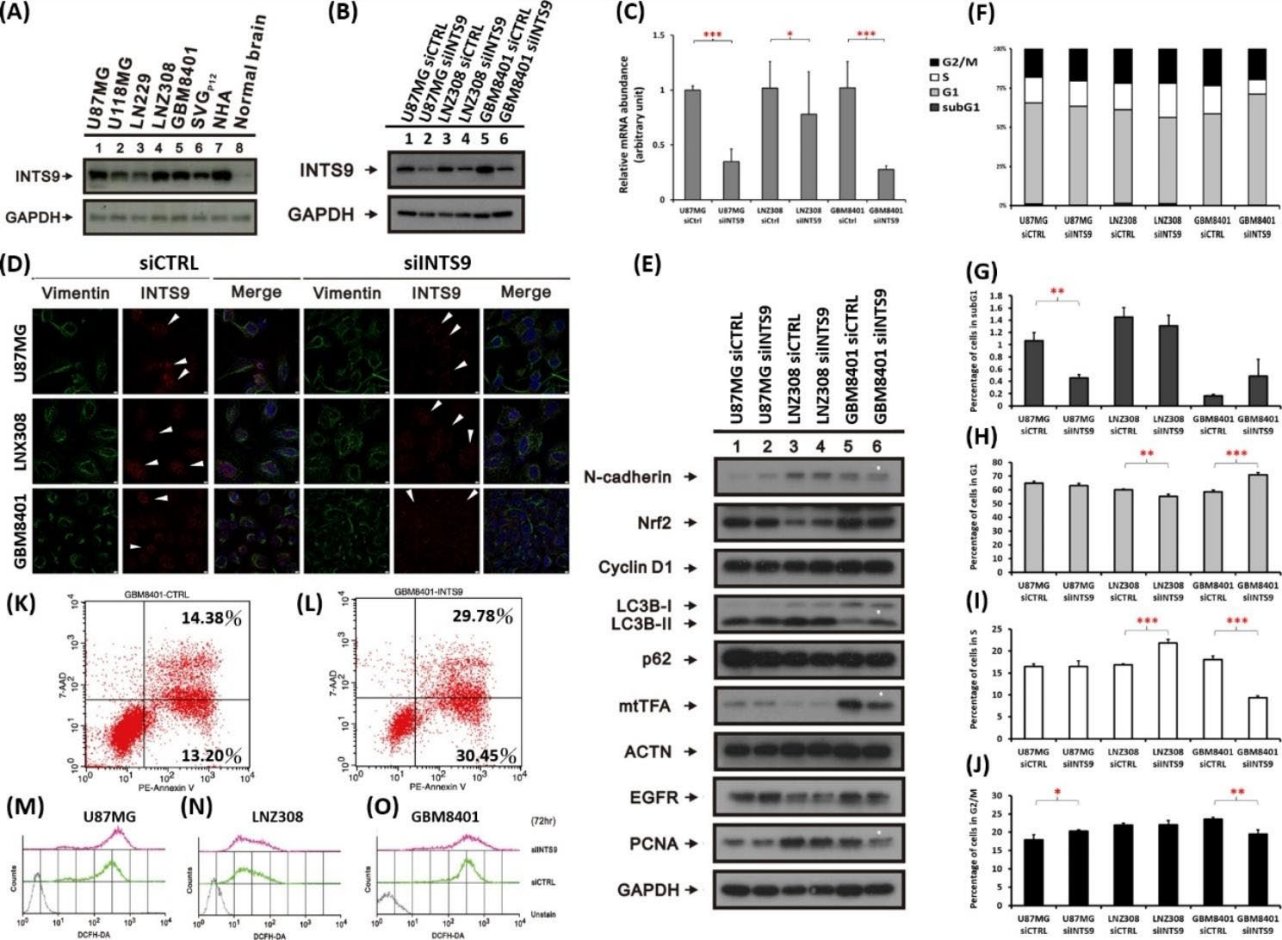
The functional validation was achieved by downregulating INTS9 expression using siINTS9. (**A**) The presence of INTS9 was ascertained in various tumor cell lines, with increased expression compared to normal brain lysates. (**B-C**) After siINTS9 administration, INTS9 expression decreased at protein and transcriptomic levels. (**D**) Decreased INTS9 was identified after being supplemented with siINTS9, and the arrowhead indicated tumor cells. (**E**) Investigated cancer hallmark-related and proliferation markers; notably, GBM8401 cells showed reduced mtTFA and N-cadherin but increased LC3BII and PCNA, highlighted with asterisk symbols. (**F-J**) Cell cycle analysis revealed a significant decrease in the S phase and G2/M in GBM8401 cells, with a slight increase in the sub-G1 phase. (**K-L**) Compared to the control group, an increased apoptosis proportion in the GBM8401 cell line when supplemented with siINTS9. (**M-O**) However, no noticeable change in cytosolic ROS was observed in the tested cell lines. Bars, mean ± SEM; *p < 0.05, **p < 0.01, ***p < 0.001

To further explore the functional implications of INTS9, we employed siINTS9 to suppress the expression of INTS9 to examine the related phenotypic alterations. Initially, we ascertained the presence of INTS9 in various tumor cell lines, such as U87MG, U118MG, LN229, LNZ308, and GBM8401. The findings indicated an increased expression of INTS9 in tumor cells instead of normal brain lysates (Fig. 7A), corroborating previous bioinformatics investigations. Subsequently, we administered siINTS9 to higher-expressed cell lines, including U87MG, LNZ308, and GBM8401, and observed a significant reduction in INTS9 expression at both the protein (Fig. 7B) and transcriptomic levels (Fig. 7C). Immunofluorescence validation revealed diminished INTS9 expression across all three cell lines post-siINTS9 treatment (Fig. 7D and Supplementary 4). We proceeded to examine a variety of cancer hallmark-related markers, such as N-cadherin for Epithelial-mesenchymal transition (EMT), Nrf2 for protection against reactive oxygen species (ROS), mtFTA for mitochondrial biogenesis, and p62, LC3BI, and LC3BII for autophagy. Our observations revealed a decrease in mtTFA and N-cadherin, but an increase in LC3BII within the GBM8401 cell line (Fig. 7E, denoted by an asterisk), while no changes were observed in the other cell lines. Earlier GSEA analyses revealed numerous alterations in signaling pathways associated with cell proliferation. Consequently, we assessed several proliferation markers, including EGFR and Proliferating Cell Nuclear Antigen (PCNA), and conducted cell cycle analysis. We observed a significant reduction in PCNA proliferation markers in the GBM8401 cell line upon siINTS9 treatment. In the cell cycle analyses (Fig. 7F-J), we detected a considerable decrease in the S phase and G2/M in GBM8401 cells (Fig. 7I-J). Furthermore, we identified a trend of a slight increase in the sub-G1 phase in GBM8401 with borderline significance (p = 0.0577, Fig. 7G). We performed the apoptosis analysis to dissect further the increased portion of the sub-G1 phase of GBM8401 cells. When supplemented with siINTS9p, a higher proportion of apoptosis was observed than in the control group in GBM8401 (Fig. 7K-L). However, there is no apparent change in the cytosolic ROS in all tested cell lines (Fig. 7M-O).

### Result 6: Functional modeling supports that higher INTS9 levels are associated with enhanced proliferation signaling

**Fig. 8:**
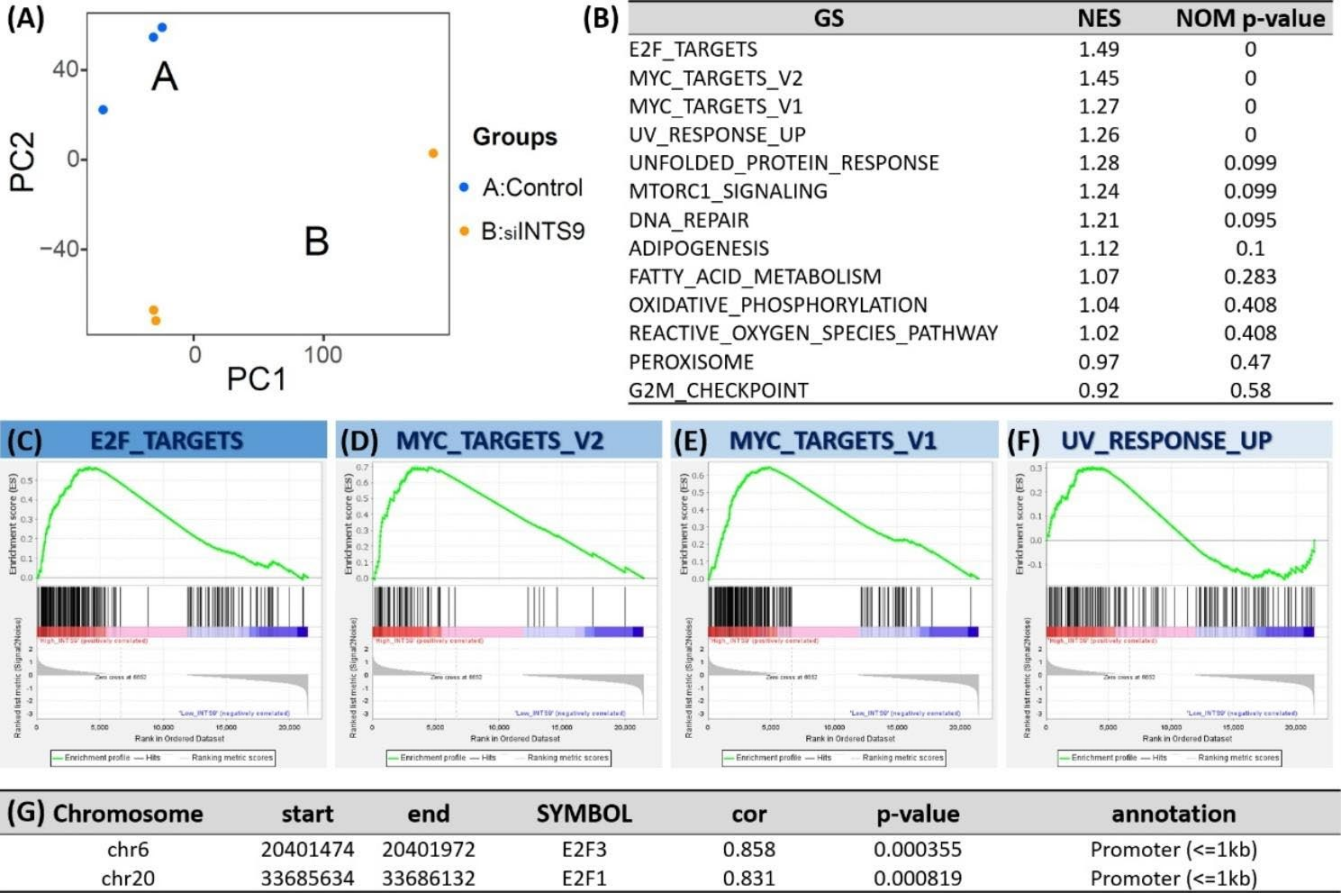
The GSEA analyses the mRNA sequencing between GBM8401 with scrambled siRNA and GBM8401 with siINTS9. (**A**) PCA plot demonstrated distinct transcriptome features between groups. (**B-F**) GSEA analysis showed significant upregulation of E2F_TARGETS, MYC_TARGETS_V1/V2, and UV_RESPONSE_UP in the higher INTS9 group. (**G**) Additionally, ATAC sequencing revealed INTS9 expression linked to chromatin accessibility of E2F1 and E2F3

To corroborate the GSEA findings and prior functional models suggesting INTS9’s involvement in proliferation signaling, we executed comprehensive mRNA sequencing, comparing the GBM8401 control group (higher INTS9) to GBM8401 cells supplemented with siINTS9 (lower INTS9). The processed transcriptome data is presented in (Supplementary 5). The PCA plot (Fig. 8A) revealed distinct transcriptome features, as control samples formed a separate cluster from the siINTS9-treated group. GSEA analysis (Fig. 8B-F) demonstrated a significant increase in E2F_TARGETS, MYC_TARGETS_V1/V2, and UV_RESPONSE_UP for the high INTS9 group. The first three gene sets correlated with proliferation capability, and although G2M lacked statistical significance, it also appeared at the top of the list after analysis. These findings from cell line models aligned with earlier GSEA analyses from TCGA datasets. Additionally, ATAC sequencing indicated a connection between INTS9 expression and chromatin accessibility of E2F1 and E2F3 (Fig. 8G and Supplementary 6).

### Result 7: In addition to the tumor cells, the expression of INTS9 was also influenced by myeloid and T cell lineages

**Fig. 9:**
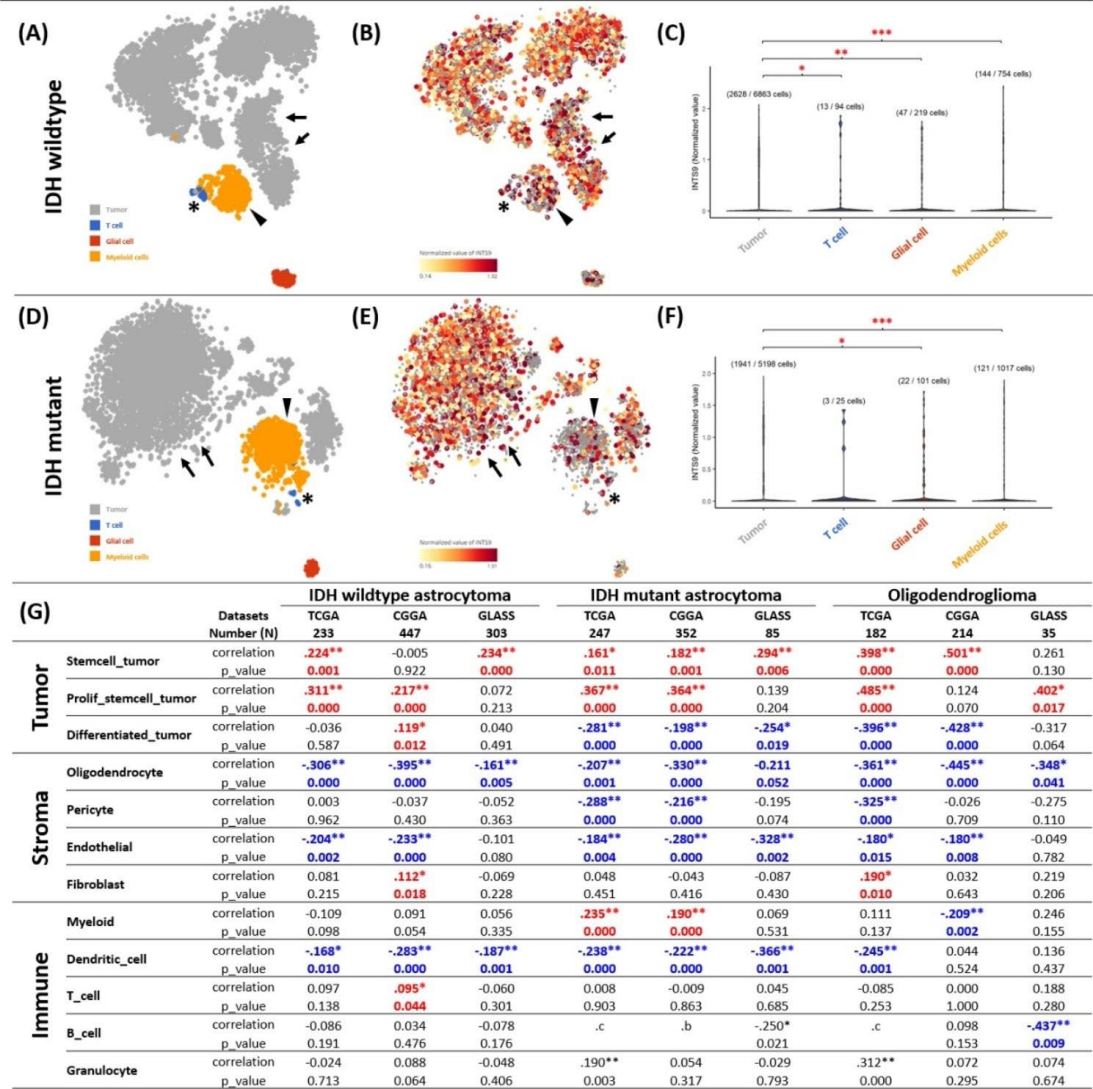
The INTS9 expression in the single-cell sequencing and 12 cell-states analyses (**A-C**) INTS9 expression strongly correlated with tumor (arrow) and myeloid lineages (arrowhead) in IDH wildtype and (**D-F**) mutant astrocytoma. The T cell also expressed high INTS9 in the IDH wildtype but equivocal in the IDH mutant group (asterisk). (**C**) Significant differences were found among tumor-to-myeloid, tumor-to-T cells, and tumor-to-reactive glia. (**F**) In IDH mutant astrocytoma, significant differences were found between tumor-to-myeloid and tumor-to-reactive glia. (**G**) INTS9 positively correlated with tumor-stem and tumor-proliferative-stem cells across all subtypes. A slight difference among subtypes is the contrasting correlation between INTS9 and differentiated tumor cells in IDH mutant and wildtype groups. INTS9 displayed a negative relationship with specific stromal cells across all three subtypes but a positive correlation with fibroblasts only in IDH wildtype and oligodendroglioma. For the immune cells, INTS9 positively correlated with myeloid cells, while a significant association with T cell quantities appeared only in IDH wildtype astrocytoma

Earlier bioinformatics characterization and functional validation of INTS9 revealed a correlation between tumor grade and proliferative function. However, certain cell lines displayed no discernible phenotypic alterations when treated with siINTS9. Furthermore, the inflammatory signals were also observed in the GSEA analyses. Consequently, we suspect the possibility of other factors associated with INTS9 that may influence prognosis. Most preceding analyses relied on bulk RNA sequencing from the tumor, which encompasses a diverse cellular heterogeneity, including tumor cells, immune cells, stromal cells, and endothelial cells. To examine the potential impact of tumor heterogeneity on INTS9 expression, we analyzed two single-cell sequencing datasets, GSE131928 and GSE89567, which represent IDH wildtype and IDH mutant astrocytoma, respectively. The heatmap of INTS9 expression demonstrated a strong correlation with tumor and myeloid lineages in both IDH wildtype (Fig. 9A-C) and IDH mutant astrocytoma (Fig. 9D-E). We also noticed a marked increase of INTS9 in the T cell population in the IDH wildtype but equivocal in the IDH mutant group. Significant differences were noted between tumor-to-myeloid, tumor-to-T cells, and tumor-to-reactive glia, with p-values of < 0.001, < 0.05, and < 0.01, respectively. (Fig. 9C). In IDH mutant astrocytoma, significant differences were found between tumor-to-myeloid and tumor-to-reactive glia, with respective p-values of < 0.001 and < 0.05. (Fig. 9F). In order to dissect the association among INTS9, myeloid cells, T cells, and additional cellular elements, we deconvoluted the bulk transcriptomes into 12 essential cellular states as defined by Prof. Roel G W Verhaak’s team [34]. Within the tumor components, we observed a positive correlation between INTS9 and tumor-stem cells and tumor-proliferative-stem cells across all three subtypes and various databases (Fig. 9G). This finding is consistent with prior experimental validation indicating an association between INTS9 and cellular proliferation. A notable distinction among these subtypes is the inverse correlation between INTS9 and differentiated tumor cells in IDH mutant astrocytoma and oligodendroglioma; conversely, a positive correlation was identified in the IDH wildtype group. Regarding the stromal cell components, INTS9 exhibited a trend of a negative association with oligodendrocytes, pericytes, and endothelial cells across all three subtypes but a positive correlation with fibroblasts, particularly in the IDH wildtype and oligodendroglioma. In the immune cell components, we observed a positive correlation between myeloid cells and INTS9 expression in mutant astrocytoma but reversely associated with the dendritic cells in all subtypes (Fig. 9G). In contrast, a significant association with T cell quantities was only evident in the IDH wildtype astrocytoma. These findings align with the results of the single-cell sequencing analyses (Fig. 9A-B, asterisk).

### Result 8: Distinct associations of INTS9 with immune cell populations and T Cell function in IDH wildtype and mutant gliomas

In the previous sections, we observed a correlation between INTS9 and inflammatory cells, including myeloid and T cells. To further elucidate this relationship with immune cells, we employed the CIBERSORT algorithm Alizadeh Lab [29] developed to predict the presence of 22 distinct resistant cell types for subsequent investigation involving INTS9. Our findings indicated that INTS9 was significantly associated with increased M0 macrophages and monocyte reduction in both IDH wildtype astrocytoma and oligodendroglioma (Table 1). In contrast, within IDH mutant astrocytoma, INTS9 demonstrated a significant correlation with M2 macrophages but no decrease in monocytes compared to other groups. Moreover, we identified an enhancement in T cell function, encompassing elevated levels of cytotoxic T cells (CD8 T cells), CD4 T memory-activated cells, follicular T cells, and regulatory T cells, exclusively in the IDH wildtype group, but not in the other groups (Table 1). These results, revealing an increase in T-cell function limited to the IDH wildtype group, align with previous discoveries from single-cell analyses (Fig. 9A-B, asterisk).

**Table 1:**
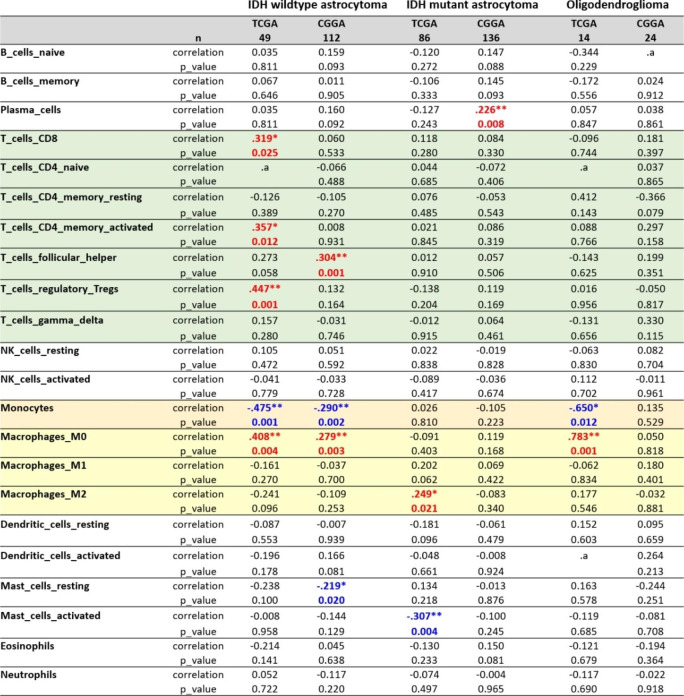
The correlation with INTS9 expression with 22 different immune cells through deconvoluted bulk transcriptome from TCGA and CGGA datasets. Our results revealed that INTS9 was significantly correlated with increased M0 macrophages and reduced monocytes in IDH wildtype astrocytoma and oligodendroglioma. In IDH mutant astrocytoma, INTS9 was associated with M2 macrophages without monocyte reduction. Enhanced T cell function, including elevated cytotoxic T cells, CD4 T memory-activated cells, follicular T cells, and regulatory T cells, was exclusive to the IDH wildtype group

### Result 9: The INTS9 expression dropped during recurrence in the IDH wildtype glioma

**Fig. 10:**
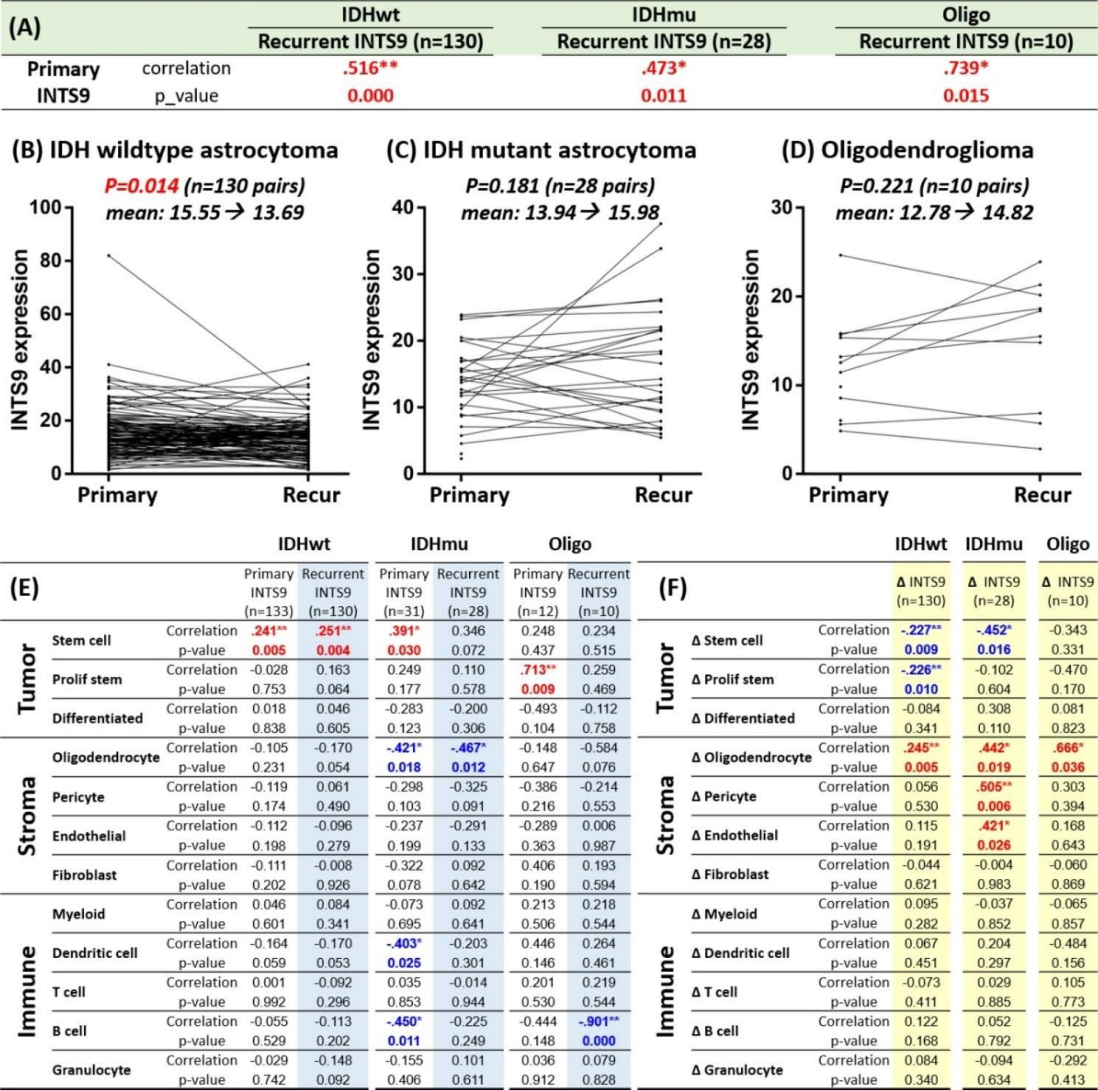
Analysis of INTS9 expression in the Glioma Longitudinal AnalySiS (GLASS) dataset. (**A**) Comparing primary and recurrent INTS9 expression revealed a moderate-to-high correlation in all subgroups. (**B-D**) INTS9 significantly decreased during recurrence in IDH wildtype, with no difference in other groups. (**E**) We noticed a positive association between INTS9 expression and tumor stem and proliferative stem cells component. (**F**) The decline in INTS9 correlated with reduced tumor stem cells and proliferative tumor stem cells in IDH wildtype and only decreased tumor stem cells in IDH mutant astrocytoma. Additionally, the decrease in INTS9 is positively associated with an increase in oligodendrocytes, pericytes, and endothelial cells, which are low in INTS9 expression compared to the tumor or myeloid lineages, which highly express INTS9

Subsequently, we examined the dynamic alterations in INTS9 between primary and recurrent tumors utilizing the Glioma Longitudinal AnalySiS (GLASS) dataset, which comprises 423 samples, including 168 paired studies (130 IDH wildtype, 28 IDH mutant, and 10 oligodendrogliomas). We initially compared primary versus recurrent INTS9 expression across the three subtypes. We observed a moderate-to-high correlation between primary and recurrent lesions (Fig. 10A), suggesting that INTS9 levels were inherited from the initial state. Upon comparing INTS9 changes between primary and recurrent tumors, we found a significant decrease in INTS9 during recurrence in IDH wildtype (Fig. 10B), whereas no significant difference was detected in the other two groups (Fig. 10C, D). In the expression data compared to the 12 cell states, we observed a positive association between INTS9 expression and tumor stem & proliferative stem cells (Fig. 10E), consistent with previous analyses using TCGA and CGGA datasets (Fig. 9G). To uncover potential reasons for the decline in INTS9, we correlated the difference between primary and recurrent INTS9 (∆_INTS9 = recurrent INTS9 – primary INTS9) with the difference in each cell state. We found that the reduction in INTS9 was linked to a decrease in tumor stem cells and proliferative tumor stem cells in IDH wildtype and only correlated with a decline in tumor stem cells in IDH mutant astrocytomas (Fig. 10F). Additionally, the decrease in INTS9 was positively associated with an increase in oligodendrocytes and occasionally with pericytes and endothelial cells (Fig. 10F), which are the INTS9 cold cellular components, contrasting with the tumor and myeloid lineage cells that exhibit high INTS9 expression (Fig. 9C, F).

## Discussion

In contemporary times, the diagnostic capabilities for brain tumors have advanced significantly; however, treatment modalities remain restricted. Consequently, the identification of potential therapeutic targets remains a pressing medical necessity. In order to identify novel biomarkers, it was necessary to reclassify the analyzed cases based on the 2021 system instead of datasets that conformed to the 2007 classification. Furthermore, a prospective candidate should demonstrate considerable prognostic value independent of well-established factors such as age, gender, and tumor grade. Utilizing sophisticated screening approaches, the INTS9 has emerged as a promising candidate for glioblastoma therapeutics. INTS9 encodes a component of the Integrator complex, which comprises a set of proteins that interact with the C-terminal domain of RNA polymerase II. This interaction enables endonucleolytic cleavage of primary small nuclear RNA (snRNA) transcripts within the nucleus, an essential step in generating the 3’ termini of snRNA molecules [24]. This process supports the function of the spliceosome, a cellular machinery crucial for the accurate splicing of precursor messenger RNA (pre-mRNA) molecules [25]. This action is vital for producing mature messenger RNA (mRNA) that ultimately translates into functional proteins [25].

The Integrator complex has been found to exhibit relatively infrequent mutations in human malignancies, which initially hinder the recognition of its role in tumorigenesis [25]. However, some studies have recently revealed its involvement in tumor development [25], including INTS3 [35], INTS6 [36–42], INST7 [43], and INSTS8 [44–46]. The association of these integrators and tumorgenesis were sorted and summarized in (Table 2). Generally, INTS6 acts as a tumor suppressor, with their Loss of function potentially leading to tumorigenesis. Conversely, INTS7 and INTS8 exhibit oncogenic properties and may stimulate tumor growth, migration, and invasion [25]. While INTS3 showed both characteristics and revealed tumor suppression in hematology malignancy [47] but turned to an oncogene in the solid organ malignancy [48]. However, no information has been available linking INTS9 with any cancer type.

**Table 2:** Integrator’s role in tumorigenesis

Subunit	Cancer type	Role in the Tumorigenesis	Refs	
INTS3	LEUK	Loss of INTS3 → tumorigenesis	[47]	
	HCC	Increased INTS3 → tumorigenesis.	[48]	
INTS6	LUAD	Loss of INTS6 → tumorigenesis	[36, 37]	
	ESCA		[38]	
	PRAD		[39, 40]	
	ALL		[41]	
	HCC		[42]	
INTS7	LUAD	Increased INTS7→ poor prognosis. Tumor proliferation↑, migration↑, invasion↑ Tumor apoptosis↓	[43]	
	PAAD	INTS7 mutation → cancer driver	[49]	
INTS8	STAD	Increased INTS8 → poor prognosis.	[46]	
	PTCL	INTS8 mutation → cancer driver	[44]	
	HCC	Increased INTS8 → poor prognosis. Epithelial-to-mesenchymal transition↑ Metastatic potential↑	[50]	
	CHOL	Increased INTS8 → poor prognosis. Tumor apoptosis↓	[45]	

* ALL = Acute lymphoblastic leukemia; AML = Acute lymphoblastic leukemia; CHOL = Cholangiocarcinoma; ESCA = Esophageal carcinoma; HCC = hepatocellular carcinoma; LEUK = Leukemia; LUAD = Lung adenocarcinoma; PAAD = Pancreatic adenocarcinoma; PRAD = Prostate adenocarcinoma; PTCL = Peripheral T cell lymphoma; STAD = stomach adenocarcinoma

Our results found that increased INTS9 is associated with higher tumor proliferation, tumor grade, and poor prognosis in glioma. From the GSEA analyses, we identified the higher INST9 group linked with gene sets representative of the cell proliferation properties again, particularly for the E2F signaling. These findings were further supported by the association with 12-cell states (proliferative stem tumor cells) and the functional experiments by comparing the transcriptome between siControl versus siINTS9 group in GBM8401. In the ATAC sequencing, we could also identify the association between the increased INTS9 and two activated E2F factors. Next, we evaluated all possible driven mutations, genomic stability (TMB, MSI), and chromosome aberration to identify the potential upstream factors. From the analyses, we noticed that TP53 was the top relevant gene associated with high INTS9 in both IDH wildtype and mutant astrocytoma from the TCGA database. The relevance between INTS9 and TP53 was also validated in the IDH wildtype group using immunohistochemistry. Although the analysis revealed no significant association in IDH mutant astrocytoma (Fig. 2F), a trend of increased INTS9 was observed in the P53 mutant group. The potential explanations for this include the limited cases of IDH mutant glioma (Fig. 2A) and the suboptimal accuracy using immunohistochemistry for predicting TP53 status [51].

Under standard conditions, the TP53 protein functions as a critical cell cycle checkpoint, impeding the propagation of cells exhibiting DNA damage or other irregularities. Upon detecting such anomalies, TP53 can arrest the cell cycle, providing an opportunity for repair mechanisms to correct the damage or, if required, initiate apoptosis to eradicate the affected cell. This protective capacity of TP53 is vital for preserving genomic stability [52]. Nevertheless, when TP53 undergoes mutation, its capacity to regulate the cell cycle becomes impaired and may even acquire oncogenic characteristics, fostering cell survival and proliferation. Consequently, this malfunctioning TP53 may inadvertently permit damaged cells to circumvent typical checkpoints and persist in dividing [52]. The correlation between mutant TP53 and elevated INTS9 levels can be elucidated by the altered regulation of gene expression resulting from TP53 dysfunction. The INTS9 protein serves a pivotal function in processing small nuclear RNA (snRNA) molecules implicated in mRNA splicing, a process indispensable for the production of functional proteins, including those involved in the cell cycle [25], such as E2F, which facilitates cell cycle progression by activating genes essential for DNA replication and cell division. However, in the presence of mutant TP53, the standard regulatory mechanisms that constrain E2F activity may be abolished or reduced, leading to elevated E2F levels and uncontrolled cell cycle progression. Based on our ATAC sequencing analysis outcomes, we observed an enhancement in chromatin accessibility for E2F1 and E2F3 when INTS9 expression was upregulated (Fig. 8G). Moreover, during the functional validation, we demonstrated that the downregulation of INTS9 impacted E2F signaling (Fig. 8B-C).

Apart from the mutant TP53 that might contribute to the increased INTS9, we also noticed that the chromosome aberrations play roles here. Our results showed that the combined chromosome 7 gain/10 loss and TERT promoter mutation associated with high INTS9 in the IDH wildtype astrocytoma during the CDKN2A/CDKN2B homozygous deletion in the IDH mutant glioma. The gain of chromosome 7 / loss of chromosome 10 and TERT promoter mutation are frequently observed in glioblastoma multiforme (GBM) genetic alterations and have been linked with highly aggressive glioma [9]. CDKN2A and CDKN2B are tumor suppressor genes that regulate cell cycle progression [53]. Homologous deletion of these genes leads to the loss of their function, promoting uncontrolled cell proliferation and contributing to the development and progression of cancer [53]. Interestingly, the presence of homozygous deletions of CDKN2A/B directly impacts the prognosis, which association has also been established in the IDH mutant astrocytoma recently [5].

In addition to the increased cellular proliferation, we also noticed that increased INTS9 was relevant to the changes in immune microenvironments, including the increased macrophage, T cell function, and decreased monocytes. Brain tumors, particularly glioblastomas, often have more macrophages [54], known as tumor-associated macrophages (TAMs). TAMs are attracted to the tumor site by various chemokines and cytokines released by the tumor cells and the surrounding stromal cells. Mutant TP53 may contribute to an immunosuppressive microenvironment that supports TAM recruitment and polarization towards an M0, M1, or M2 phenotype, which promotes tumor growth, angiogenesis, and immune evasion. The decrease in monocytes observed in brain tumors may result from their differentiation into macrophages upon entering the tumor microenvironment [54]. Moreover, TP53 mutations could instigate T-cell activation, acting as part of the host’s immune response against the tumor [52]. However, based on our findings (Table 1), T cell lineage activation is solely observed in IDH wildtype astrocytoma and not in its IDH mutant counterpart. This inconsistency might be attributed to the disparate microenvironments in the two subgroups [55]. Recent work by Michael Platten’s team has underscored that the IDH mutation exerts stringent control over the state of intra-tumoral myeloid cells [55]. Specifically, IDH-mutant astrocytoma obstructs regular myeloid cell differentiation while instigating a T cell-suppressive milieu via monocyte-derived macrophages [55]. This IDH mutation also alters macrophage tryptophan metabolism, impacting adjacent cells [55]. This phenomenon explains the immune activation uniquely observed in the IDH wildtype astrocytoma but not IDH mutant.

The longitudinal analyses of the correlation between INTS9 and the three main tumor components within the GLASS dataset show some inconsistencies between initial and recurring samples (Fig. 10E**)**. In the IDH-mutant astrocytoma, despite not reaching statistical significance between INTS9 with the fraction of recurrent tumor stem cells, however, a trend of association was noted, with a p-value of 0.072, nearing the accepted threshold of 0.05. It could be due to the reduced sample size caused by classifying cases into primary and recurrent groups compared to investigating them together, as shown in (Fig. 9G**)**. Moreover, in the oligodendroglioma group, a correlation between INTS9 and the tumor proliferative stem cell component was only observed in primary tumors, not recurrences, possibly due to the limited sample size (n = 10) and significant tumor heterogeneity. Recurrent tumors tend to have more mixed tissue composition, including scar tissue, normal components from broader surgical removal, and increased post-surgical immune responses. These aspects could collectively influence INTS9 expressions, leading to a more complex interaction with various potential variables than the primary tumor state.

In glioblastoma, cancer stem cells (CSCs) have been observed to resist anti-proliferative treatments, such as Temozolomide [56]. Upon completion of chemotherapy, these CSCs re-emerge, potentially fostering tumor growth and leading to a lack of successful treatment response [56]. Developing new therapeutic approaches targeting CSCs could potentially delay tumor recurrence. Our research indicates a robust correlation between INTS9, tumor stem cells, and proliferative states within the tumor. As a result, directing therapeutic strategies toward INTS9 could influence these cell populations and extend survival across all glioma subtypes, despite the IDH status. However, the influence of IDH status on alterations in the extracellular matrix and metabolism, which significantly impacts immune responses [55], must be considered. Therefore, the implementation of combined immunotherapy may fluctuate among different subtypes. In-depth, comprehensive research is still crucial and needed for determining the optimal therapeutic strategy for patients diagnosed with brain cancer.

## Conclusion

This investigation represents the inaugural study to uncover the involvement of INTS9 in glioma through the integration of comprehensive genomic (TCGA, CGGA), longitudinal analyses, single-cell, ATAC-sequencing datasets, and functional validation. The findings suggest that augmented INTS9 expression is associated with heightened proliferative capacity, high tumor grading, diminished prognosis, and the influence of the neoplastic microenvironment on immune cell interactions, potentially attributable to TP53 mutations. The concept map of this study is demonstrated in (Supplementary 7) and sheds light on the potential role of INTS9, presenting a perspective of therapeutic value for clinical application.

### Electronic supplementary material

Below is the link to the electronic supplementary material.

Supplementary 1: Case summary in bioinformatics & immunohistochemistry

Supplementary 2: Mutation matrix of 892 GBMLGG cases

Supplementary 3: Immunohistochemistry quantification for INTS9, IDH, P53, & KI67

Supplementary 4: Immunofluorescence of INTS9 with or without siRNA targeting

Supplementary 5: Transcriptomes (TPM) of GBM8401 with or without siINTS9

Supplementary 6: Result of ATAC sequencing analysis

Supplementary 7: Concept map of current study

Supplementary 8: Original data of Western-Blot for Fig. 7

## Data Availability

Datasets used in this project can be accessed via the TCGA GDC portal (https://portal.gdc.cancer.gov/), CGGA (http://www.cgga.org.cn/), GLASS (https://www.synapse.org/#! Synapse:syn17038081/ wiki/585,622) and the Single-cell sequencing data from Gene-Expression Omnibus (GEO) databases, GSE131928 (https://www.ncbi.nlm.nih.gov/geo/query/acc.cgi?acc=GSE131928) and GSE89567 (https://www.ncbi.nlm.nih.gov/geo/query/acc.cgi?acc=GSE89567). The Glioma ATAC sequence was from (https://gdc.cancer.gov/aboutdata/publications/ATACseq-AWG). Lastly, the raw NGS sequencing data from cell-line validation are available at GEO, GSE233640 (https://www.ncbi.nlm.nih.gov/geo/query/acc.cgi?&acc=GSE233640).

## Abbreviations

CBTRUS: Central Brain Tumor Registry of the United States
CGGA: Chinese Glioma Genome Atlas
GEO: Gene-Expression Omnibus
GLASS: Glioma Longitudinal AnalySiS
GSEA: Gene Set Enrichment Analysis
INTS9: Integrator Complex Subunit 9
MSI: Microsatellite Instability
RPKM: Reads per kilobase of exon per million
siINTS9: small interfering RNA targeting the INTS9 gene
siRNA: small interfering RNA
TCGA: The Cancer Genome Atlas
TMB: Tumor mutation burden
TPM: Transcripts per million
