# Supplementary material for: Decoding the prognostic significance of integrator complex subunit 9 (INTS9) in glioma: links to TP53 mutations, E2F signaling, and inflammatory microenvironments
Source: Cancer Cell Int. 2023 Aug 3;23:154. doi: 10.1186/s12935-023-03006-5 (PMC10401760; doi:10.1186/s12935-023-03006-5)
Supplement: Supplementary file 1 — Supplementary 1: Case summary in bioinformatics & immunohistochemistry [file 12935_2023_3006_MOESM1_ESM.docx]

**Supplementary 1.** Summary of the case number in each bioinformatic and immunohistochemistry analysis

**A.** For this study, primary mRNA analyses were conducted using independent samples obtained from the TCGA (relevant to **Figure 1**, **Figure 3**, **Figure 4**, and **Figure 5**). The raw data can be accessed via <https://portal.gdc.cancer.gov/>.

| 2007 adult glioma classification | N |  |  | 2021 adult glioma classification | N | |
| --- | --- | --- | --- | --- | --- | --- |
| Grade 2 oligodendroglioma | 115 |  | **Oligo** | Grade 2 oligodendroglioma | 183 | 101 |
| Grade 2 oligoastroctyoma | 77 |  |  | Grade 3 oligodendroglioma |  | 82 |
| Grade 2 astrocytoma | 65 |  | **IDHmu** | Grade 2 IDH mutant astrocytoma | 247 | 124 |
| Grade 3 oligodendroglioma | 82 |  |  | Grade 3 IDH mutant astrocytoma |  | 101 |
| Grade 3 oligoastroctyoma | 55 |  |  | Grade 4 IDH mutant astrocytoma |  | 22 |
| Grade 3 astrocytoma | 130 |  | **IDHwt** | Grade 2 IDH wildtype astrocytoma | 232 | 14 |
| Grade 4 glioblastoma | 166 |  |  | Grade 3 IDH wildtype astrocytoma |  | 10 |
|  |  |  |  | Grade 4 IDH wildtype astrocytoma |  | 208 |
| Total | **690** |  |  | **Total** |  | **662*** |

*28 cases could not be further classified according to the 2021 system, as there was insufficient molecular information available.

**B.** Glioma case available with ATAC sequcing data was also used to understand the association between INTS4 and chromatin accessibility (pertaining to **Figure 7G**). The raw data can be accessed at (<https://gdc.cancer.gov/about-data/publications/ATACseq-AWG>)

| TCGA-FG-A4MU-01B-31-A615-42  TCGA-DU-6395-02A-11-A644-42  TCGA-DU-5870-02A-21-A646-42  TCGA-P5-A72X-01A-31-A617-42  TCGA-DU-6407-02B-21-A645-42 |  | TCGA-W9-A837-01A-31-A617-42  TCGA-FG-A4MY-01A-31-A616-42  TCGA-F6-A8O3-01A-31-A617-42  TCGA-P5-A72W-01A-31-A617-42 |  | TCGA-P5-A735-01A-31-A617-42  TCGA-P5-A77X-01A-31-A617-42  TCGA-DB-A75K-01A-31-A646-42  TCGA-E1-A7YI-01A-31-A617-42 |
| --- | --- | --- | --- | --- |

**C.** Validation of the INTS9 protein was carried out utilizing independent samples acquired from the TCGA-PDC (pertaining to **Figure 1M**). The raw data can be accessed at <https://pdc.cancer.gov/pdc/>.

| Histology diagnosis | N |
| --- | --- |
| Grade 4 glioblastoma | 100 |
| Normal brain tissue | 10 |
| Total | 110 |

**D.** Immune cellular composition analyses were conducted using 12 cell states and CIBERSORT, drawing from an additional mRNA dataset provided by CGGA, which contained independent samples (relevant to **Table 1**). The raw data can be accessed at <http://www.cgga.org.cn/>.

| 2007 adult glioma classification | N |  |  | 2021 adult glioma classification | N | |
| --- | --- | --- | --- | --- | --- | --- |
| Grade 2 oligodendroglioma | 112 |  | **Oligo** | Grade 2 oligodendroglioma | 213 | 106 |
| Grade 2 oligoastroctyoma | 9 |  |  | Grade 3 oligodendroglioma |  | 107 |
| Grade 2 astrocytoma | 175 |  | **IDHmu** | Grade 2 IDH mutant astrocytoma | 351 | 130 |
| Grade 3 oligodendroglioma | 92 |  |  | Grade 3 IDH mutant astrocytoma |  | 141 |
| Grade 3 oligoastroctyoma | 21 |  |  | Grade 4 IDH mutant astrocytoma |  | 80 |
| Grade 3 astrocytoma | 215 |  | **IDHwt** | Grade 2 IDH wildtype astrocytoma | 444 | 53 |
| Grade 4 glioblastoma | 388 |  |  | Grade 3 IDH wildtype astrocytoma |  | 98 |
|  |  |  |  | Grade 4 IDH wildtype astrocytoma |  | 293 |
| Total | **1012** |  |  | **Total** |  | **1008** |

*4 cases could not be further categorized according to the 2021 system due to insufficient molecular information.

**E.** Longitudinal INTS9 mRNA analyses were performed using paired samples from GLASS (relevant to **Figure 9**). The raw data can be accessed at <https://www.synapse.org/#!Synapse:syn17038081/wiki/ 585622>.

|  | 2021 adult glioma classification | Sample number | Cases with paired sample |
| --- | --- | --- | --- |
| Oligo | Grade 2 oligodendroglioma | 12 | **(10 cases with paired sample)** |
|  | Grade 3 oligodendroglioma | 23 |  |
| IDHmu | Grade 2 IDH mutant astrocytoma | 27 | **(28 cases with paired sample)** |
|  | Grade 3 IDH mutant astrocytoma | 21 |  |
|  | Grade 4 IDH mutant astrocytoma | 37 |  |
| IDHwt | Grade 2 IDH wildtype astrocytoma | 1 | **(130 cases with paired sample)** |
|  | Grade 3 IDH wildtype astrocytoma | 2 |  |
|  | Grade 4 IDH wildtype astrocytoma | 300 |  |
|  |  | **423 samples** | **176 cases with paired sample** |
