# Supplementary material for: Decoding the prognostic significance of integrator complex subunit 9 (INTS9) in glioma: links to TP53 mutations, E2F signaling, and inflammatory microenvironments
Source: Cancer Cell Int. 2023 Aug 3;23:154. doi: 10.1186/s12935-023-03006-5 (PMC10401760; doi:10.1186/s12935-023-03006-5)
Supplement: Supplementary file 4 — Supplementary 4: Immunofluorescence of INTS9 with or without siRNA targeting [file 12935_2023_3006_MOESM4_ESM.pdf]

Supplementary 4. INTS9 immunofluorescence staining

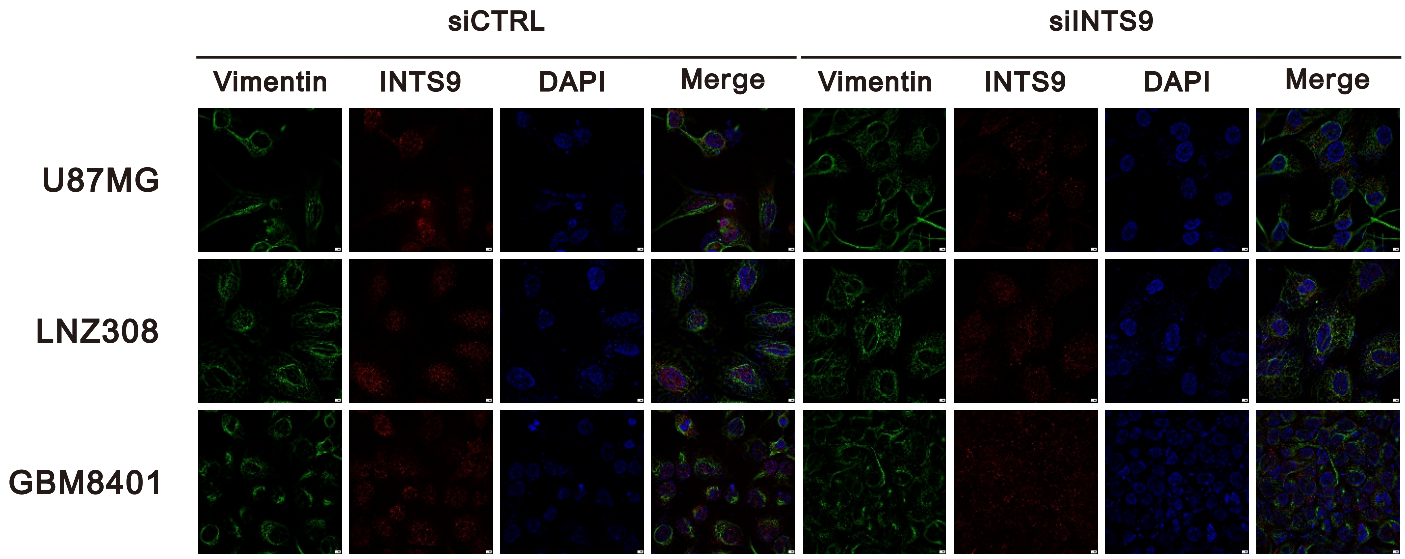

Figure legend. The use of immunofluorescence staining for INTS9 (depicted in red), Vimentin (displayed in green), and DAPI (colored blue) is shown. Vimentin was employed to emphasize the presence of the tumor cell (indicated by the arrowhead) and to analyze the expression levels of INTS9. Based on the findings, a reduction in INTS9 was observed following the introduction of siINTS9.
