# Supplementary figures and images for: Decoding the prognostic significance of integrator complex subunit 9 (INTS9) in glioma: links to TP53 mutations, E2F signaling, and inflammatory microenvironments
Source: Cancer Cell Int. 2023 Aug 3;23:154. doi: 10.1186/s12935-023-03006-5 (PMC10401760; doi:10.1186/s12935-023-03006-5)

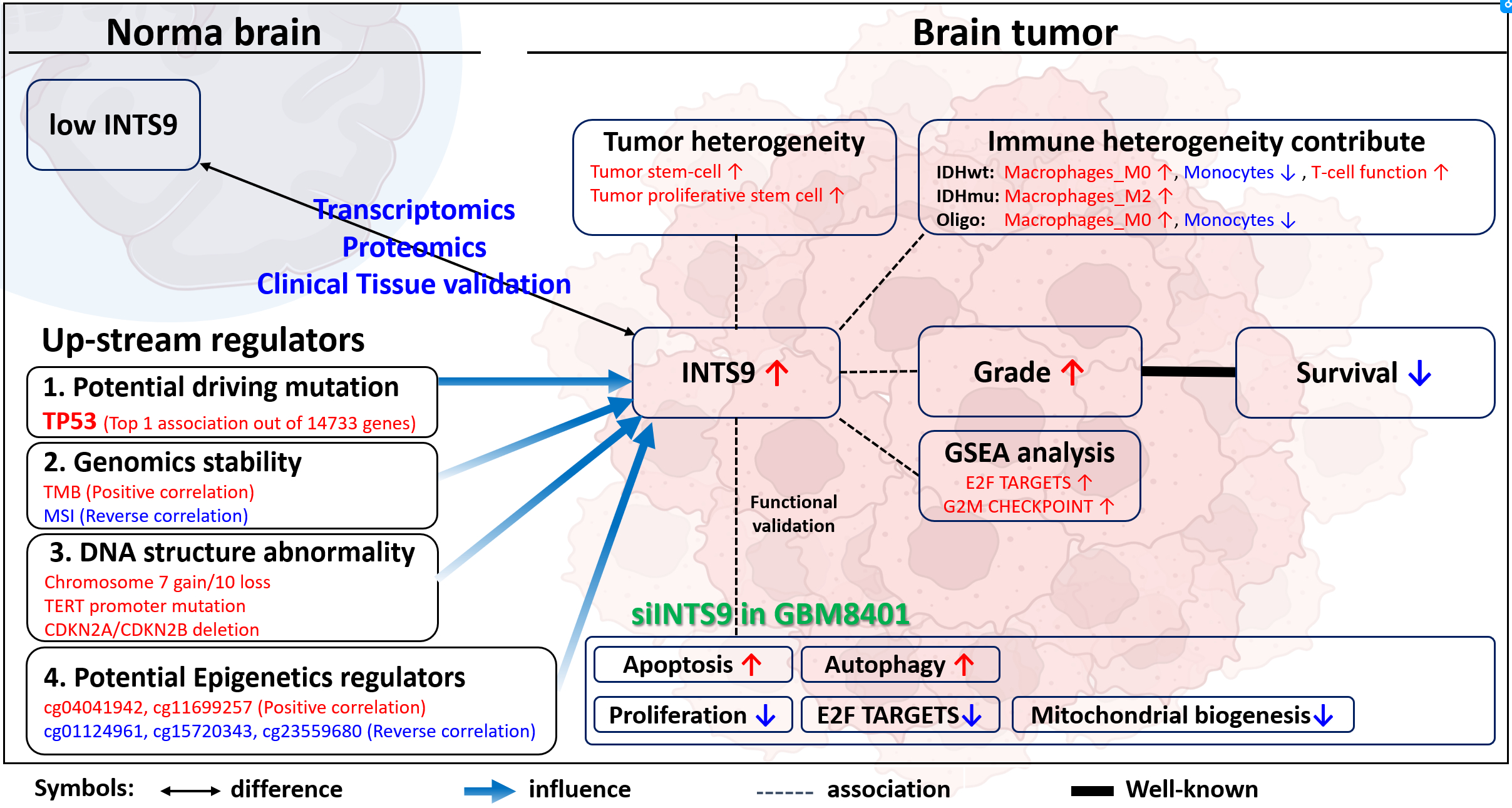

Supplement: Supplementary file 7 — Supplementary 7: Concept map of current study [file 12935_2023_3006_MOESM7_ESM.jpg]

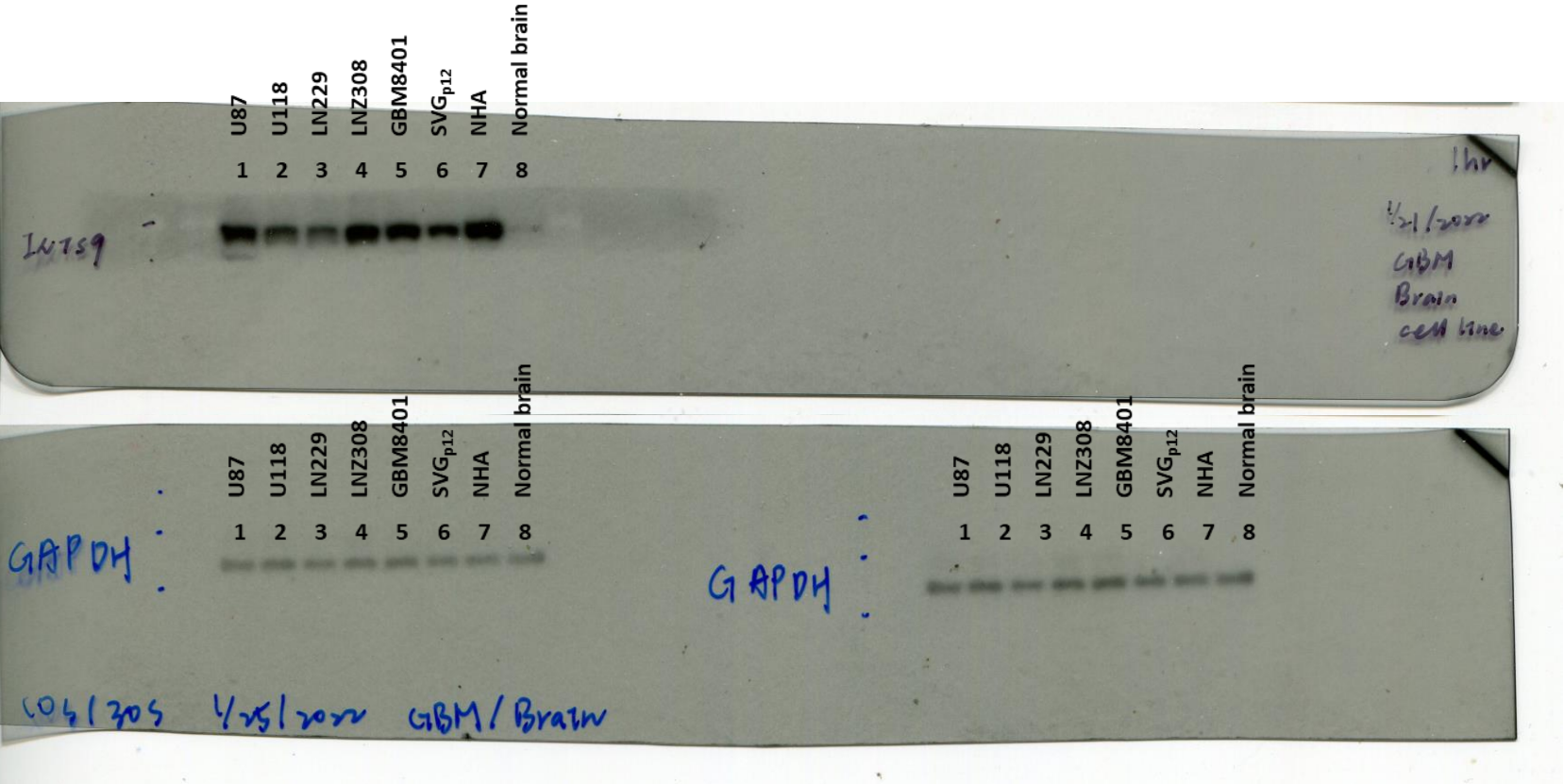

(A)

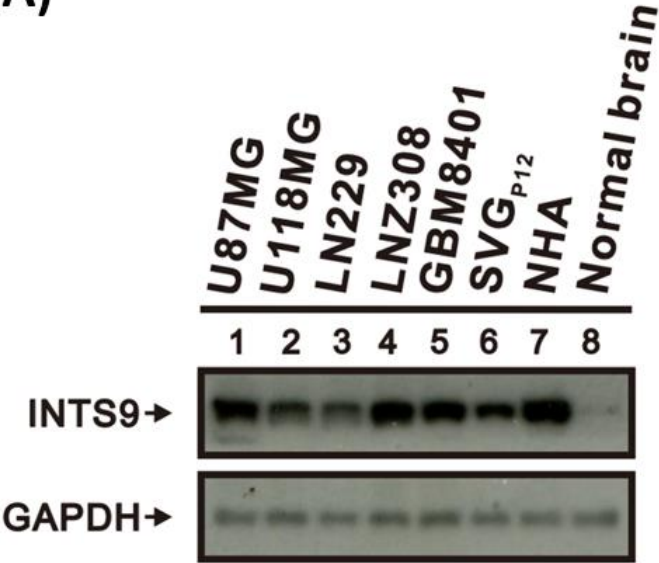

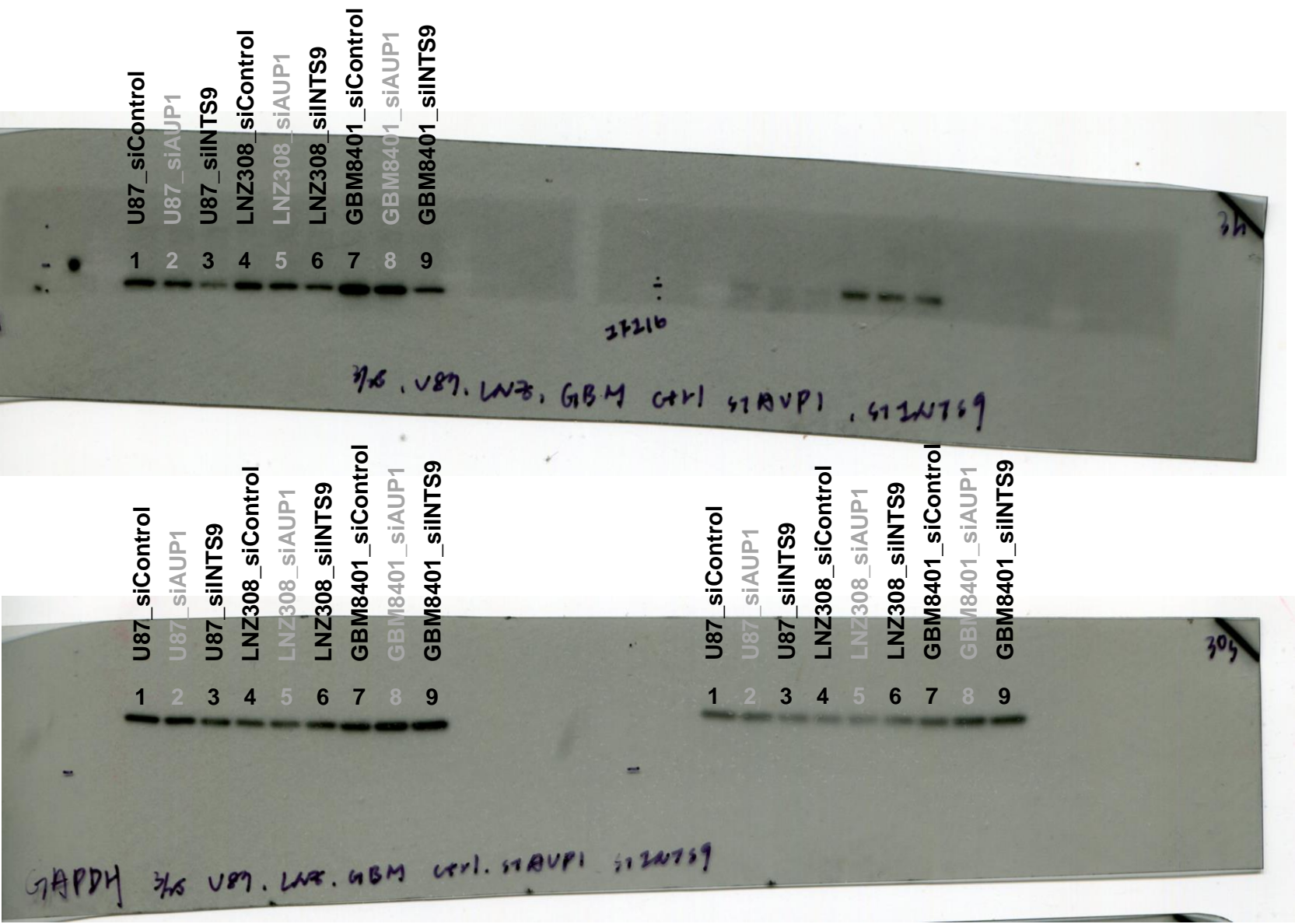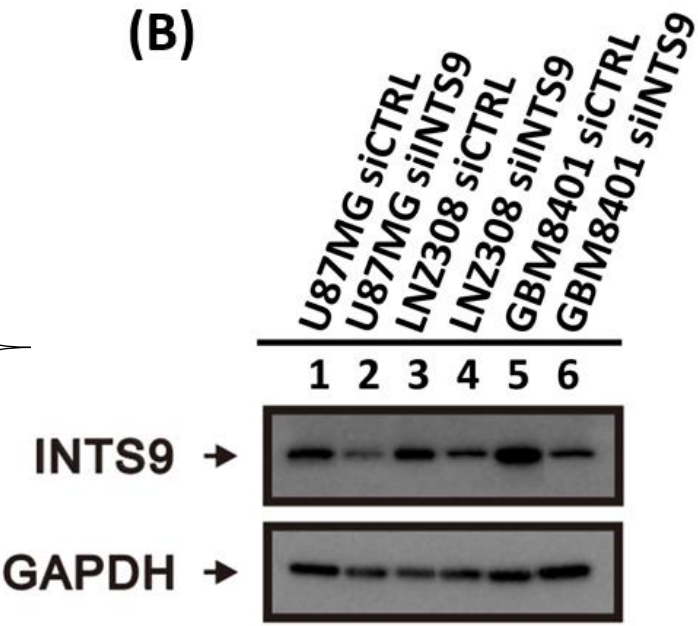

Supplementary 8\_for Figure 7(E)

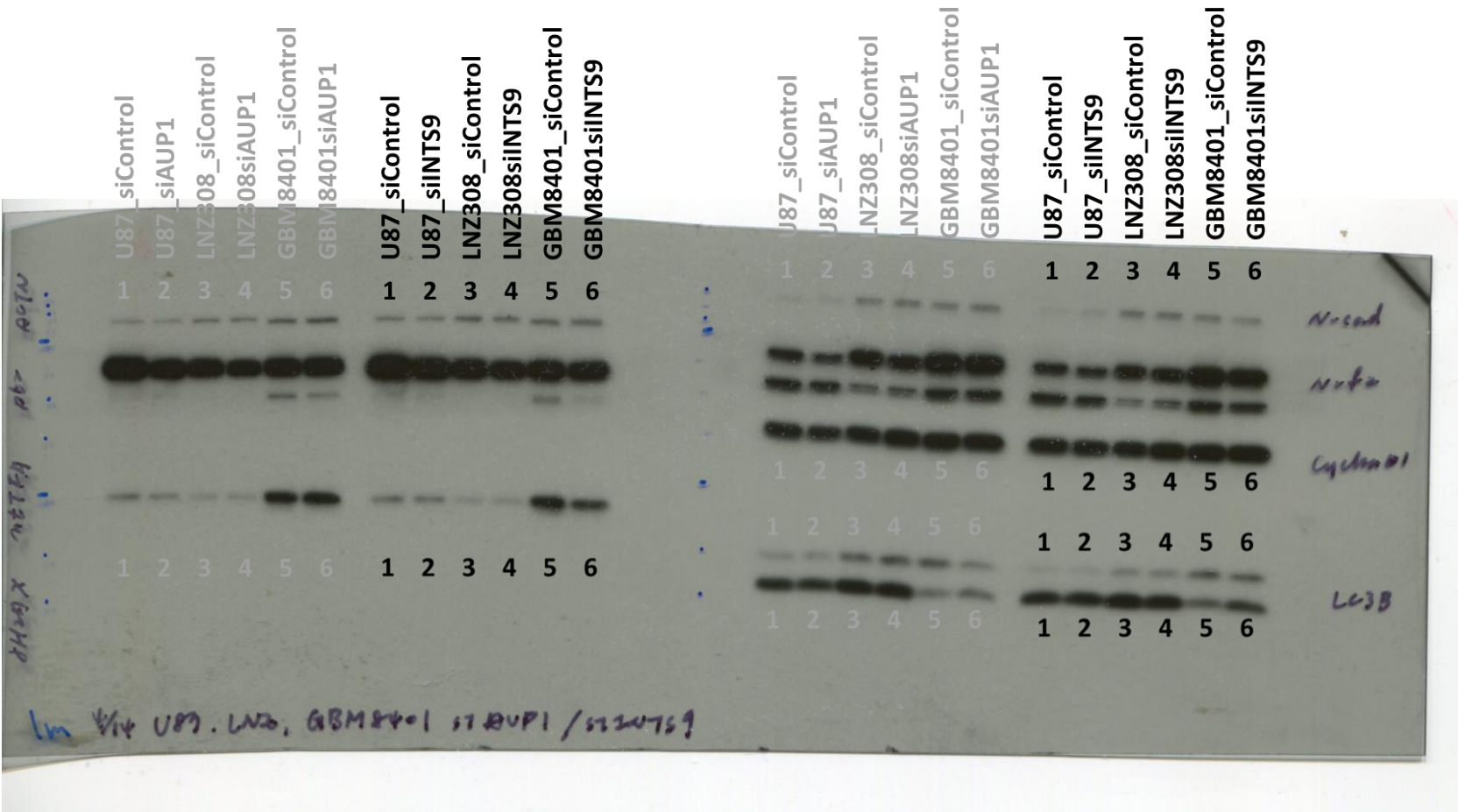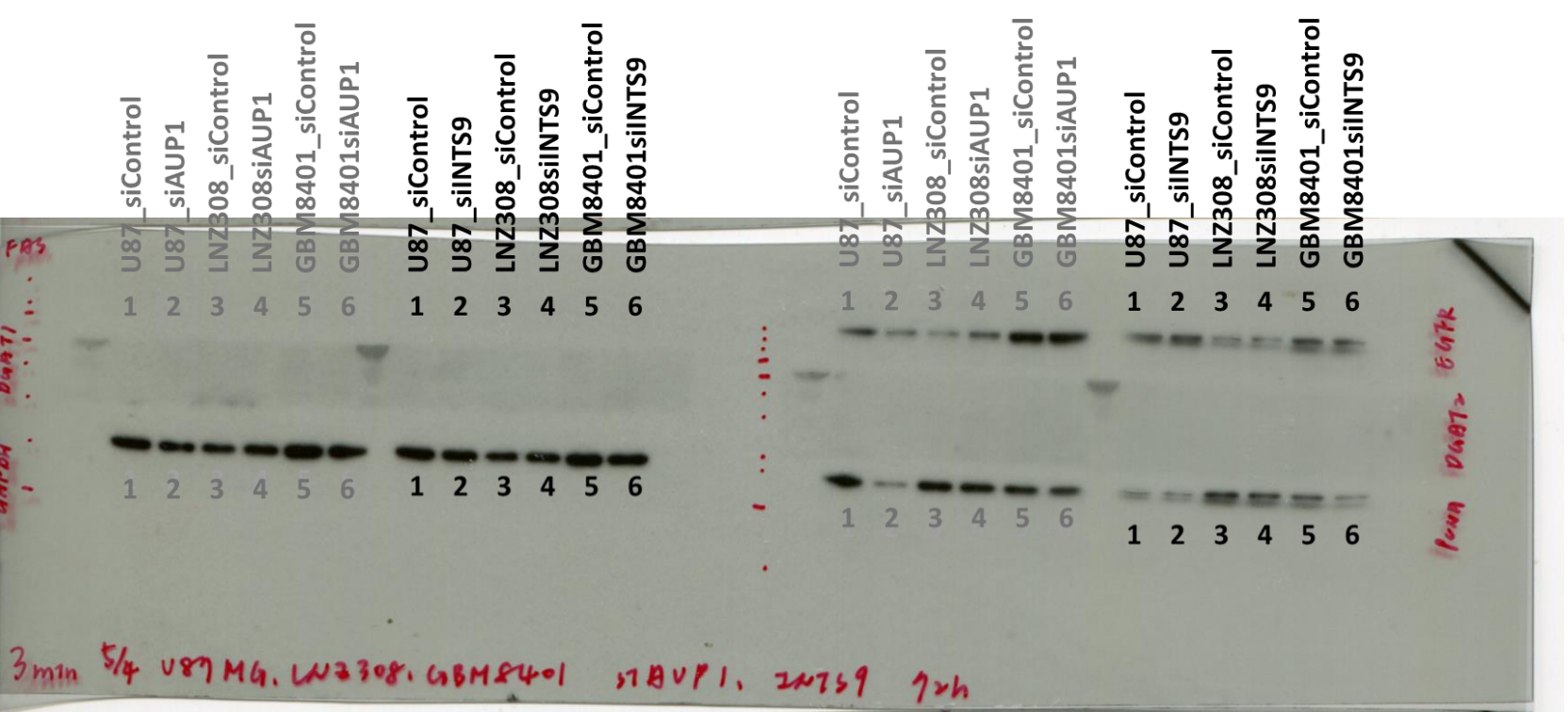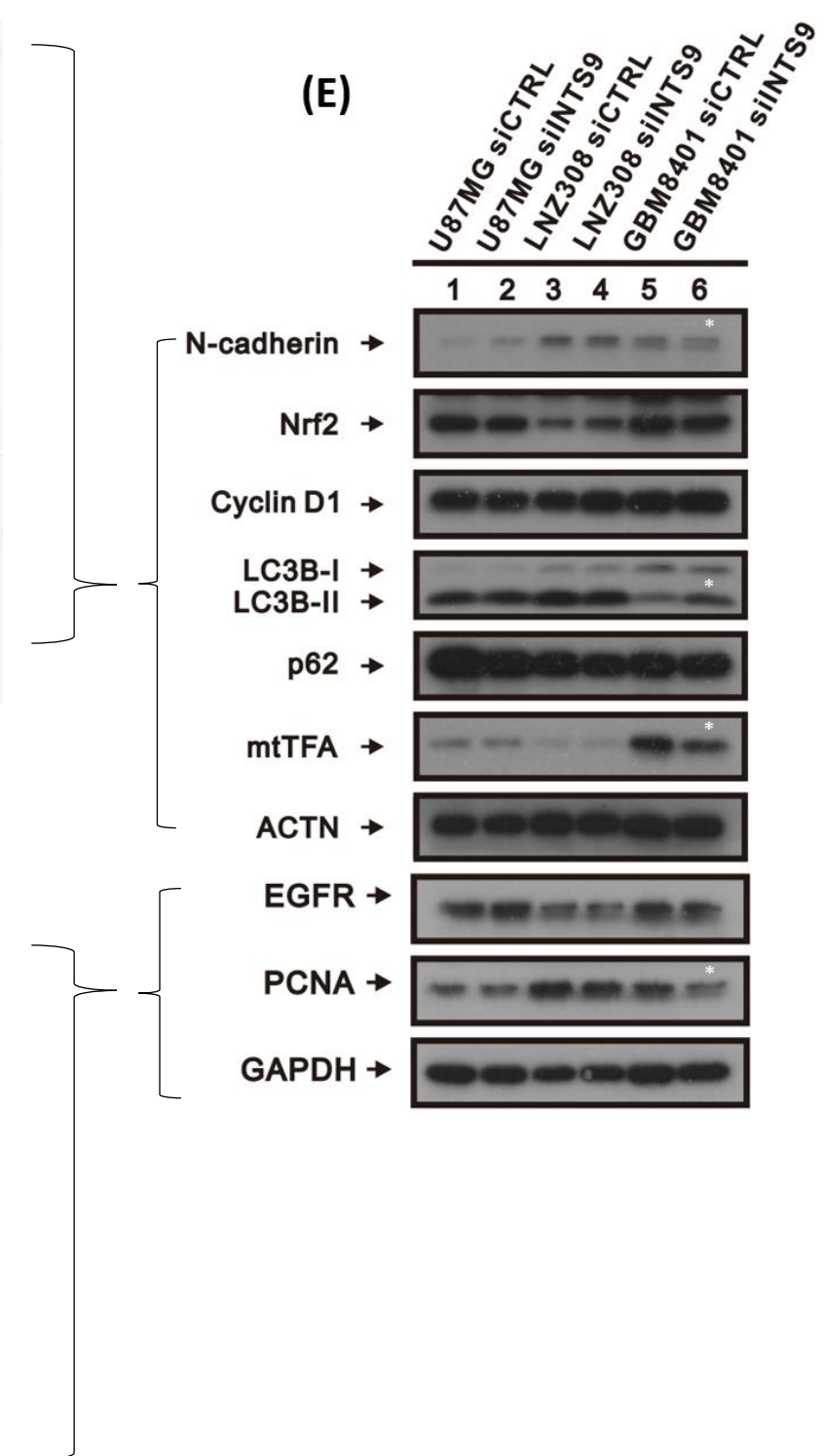

Supplement: Supplementary file 8 — Supplementary 8: Original data of Western-Blot for Fig. 7 [file 12935_2023_3006_MOESM8_ESM.pdf]
